# Using Bayes to get the most out of non-significant results

**DOI:** 10.3389/fpsyg.2014.00781

**Published:** 2014-07-29

**Authors:** Zoltan Dienes

**Affiliations:** School of Psychology and Sackler Centre for Consciousness Science, University of SussexBrighton, UK

**Keywords:** Bayes factor, confidence interval, highest density region, null hypothesis, power, statistical inference, significance testing

## Abstract

No scientific conclusion follows automatically from a statistically non-significant result, yet people routinely use non-significant results to guide conclusions about the status of theories (or the effectiveness of practices). To know whether a non-significant result counts against a theory, or if it just indicates data insensitivity, researchers must use one of: power, intervals (such as confidence or credibility intervals), or else an indicator of the relative evidence for one theory over another, such as a Bayes factor. I argue Bayes factors allow theory to be linked to data in a way that overcomes the weaknesses of the other approaches. Specifically, Bayes factors use the data themselves to determine their sensitivity in distinguishing theories (unlike power), and they make use of those aspects of a theory’s predictions that are often easiest to specify (unlike power and intervals, which require specifying the minimal interesting value in order to address theory). Bayes factors provide a coherent approach to determining whether non-significant results support a null hypothesis over a theory, or whether the data are just insensitive. They allow accepting and rejecting the null hypothesis to be put on an equal footing. Concrete examples are provided to indicate the range of application of a simple online Bayes calculator, which reveal both the strengths and weaknesses of Bayes factors.

## INTRODUCTION

Users of statistics, in disciplines from economics to sociology to biology to psychology, have had a problem. The problem is how to interpret a non-significant result. A non-significant result can mean one of two things: either that there is evidence for the null hypothesis and against a theory that predicted a difference (or relationship); or else that the data are insensitive in distinguishing the theory from the null hypothesis and nothing follows from the data at all. (Indeed, in the latter case, a non-significant result might even somewhat favor the theory, e.g., Dienes, 2008, p. 128, as will be seen in some of the examples that follow.) That is, the data might count in favor of the null and against a theory; or they might count for nothing much. The problem is that people have been choosing one of those two interpretations without a coherent reason for that choice. Thus, non-significant results have been used to count against theories when they did not (e.g., Cohen, 1990; Rosenthal, 1993; Rouder et al., 2007); or else have been ignored when they were in fact informative (e.g., believing that an apparent failure to replicate with a non-significant result is more likely to indicate noise produced by sloppy experimenters than a true null hypothesis; cf. Greenwald, 1993; Pashler and Harris, 2012; Kruschke, 2013a). One can only wonder what harm has been done to fields by not systematically determining which interpretation of a non-significant result actually holds. There are three solutions on the table for evaluating a non-significant result for a single study: (1) power; (2) interval estimates; and (3) Bayes factors (and related approaches). In this article, I will discuss the first two briefly (because readers are likely to be most familiar with them) indicating their uses and limitations; then describe how Bayes factors overcome those limitations (and what weaknesses they in turn have). The bulk of the paper will then provide detailed examples of how to interpret non-significant results using Bayes factors, while otherwise making minimal changes to current statistical practice. My aim is to clarify how to interpret non-significant results coherently, using whichever method is most suitable for the situation, in order to effectively link data to theory. I will be concentrating on a method of using Bayes that involves minor changes in adapting current practice. The changes therefore can be understood by reviewers and editors even as they operate under orthodox norms (see, e.g., Allen et al., 2014) while still solving the fundamental problem of distinguishing insensitive data from evidence for a null hypothesis.

## ILLUSTRATION OF THE PROBLEM

**FIGURE 1:**
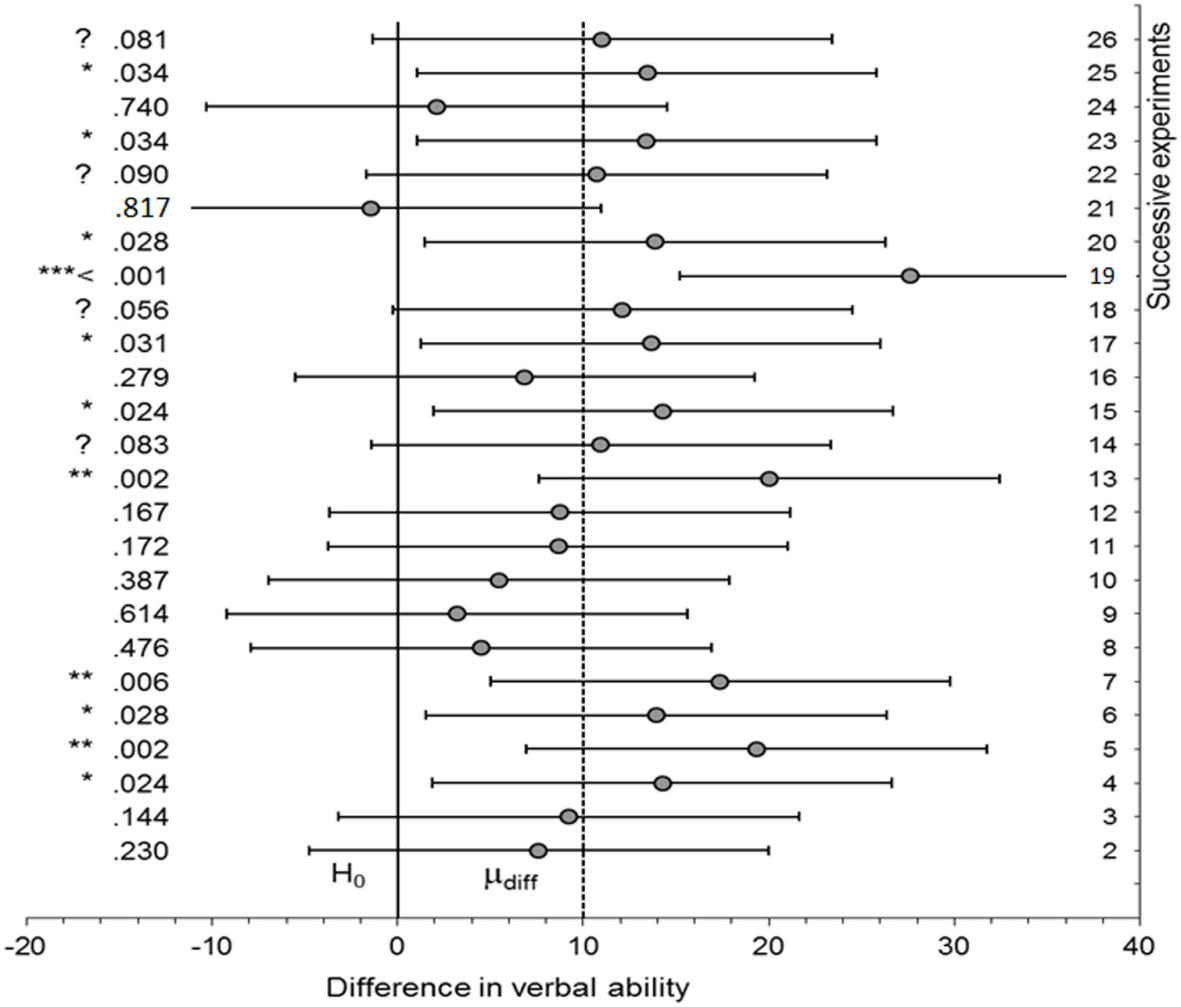
**Dance of the *p*-values.** A sequence of *p*-values (and confidence intervals) for successive simulations of an experiment where there is an actual effect (mean population difference = 10 units) and power is 0.5. Created from software provided by Cumming (2011; associated website).

Imagine that there really is an effect in the population, and the power of an experimental procedure is 0.5 (i.e., only 50 out of 100 tests would be significant when there is an actual, real effect; not that in reality we know what the real effect is, nor, therefore, what the power is for the actual population effect). The experiment is repeated exactly many times. Cumming (2011; see associated website) provides software for simulating such a situation; the use of simulation of course ensures that each simulation is identical to the last bar the vagaries of random sampling. A single run (of Cumming’s “ESCI dance *p*”) generated the sequence of *p*-values shown in **Figure 1**. Cumming (2011) calls such sequences the “dance of the *p*-values.” Notice how *p*-values can be very high or very low. For example, one could obtain a *p*-value of 0.028 (experiment 20) and in the very next attempted replication get a *p* of 0.817, when nothing had changed in terms of the population effect. There may be a temptation to think the *p* of 0.817 represents very strong evidence for the null; it is “very non-significant.” In fact, a *p*-value *per se* does not provide evidence for the null, no matter “how non-significant” it is (Fisher, 1935; Royall, 1997). A non-significant *p*-value does not distinguish evidence for the null from no evidence at all (as we shall see). That is, one cannot use a high *p*-value in itself to count against a theory that predicted a difference. A high *p*-value may simply reflect data insensitivity, a failure to distinguish the null hypothesis from the alternative because, for example, the standard error (SE) is high. Given this, how can we tell the meaning of a non-significant result?

## SOLUTIONS ON THE TABLE

### POWER

A typical orthodox solution to determining whether data are sensitive is power (Cohen, 1988). Power is the property of a decision rule defined by the long run proportion of times it leads to rejection of the null hypothesis given the null is actually false. That is, by fixing power one controls long run Type II error rates (the rate at which one accepts the null given it is false, i.e., the converse of power). Power can be easily calculated with the free software Gpower (Faul et al., 2009): To calculate *a priori* power (i.e., in advance of collecting data, which is when it should be calculated), one enters the effect size predicted in advance, a number of participants, the significance level to be used, and a power is returned.

To calculate power, in entering the effect size, one needs to know the minimal difference (relationship) below which the theory would be rendered false, irrelevant or uninteresting. If one did not use the *minimal* interesting value to calculate power one would not be controlling Type II error rates. If one used an arbitrary effect size (e.g., Cohen’s *d* of 0.5), one would be controlling error rates with respect to an arbitrary theory, and thus in no principled way controlling error rates for the theories one was evaluating. If one used a typical effect size, but not the minimal interesting effect size, type II error rates would not be controlled.

Power is an extremely useful concept. For example, imagine a review of 100 studies that looked at whether meditation improved depression. Fifty studies found statistically significant results in the right direction and 50 were non-significant. What can be concluded about the effectiveness of meditation in treating depression? One intuition is that each statistically significant result trades off against each statistically non-significant result, and nothing much follows from these data: More research is needed. This intuition is wrong because it ignores power. How many experiments out of 100 would be statistically significant at the 5% level if the null were true? Only five – that’s the meaning of a 5% significance level. (There may be a “file drawer problem” in that not all null results get published; but if all significant results were reported, and the null were true, one would expect half to be statistically significant in one direction and half in the other.) On the other hand, if an effect really did exist, and the power was 50%, how many would be statistically significant out of 100? Fifty – that’s the meaning of a power of 50%^1^ In fact, the obtained pattern of results categorically licenses the assertion that meditation improves depression (for this fictional data set).

Despite power being a very useful concept, there are two problems with power in practice. First, calculating power requires specifying the minimal interesting value, or at least the minimal interesting value that is plausible. This may be one of the hardest aspects of a theory’s predictions to specify. Second, power cannot use the data themselves in order to determine how sensitively those very data distinguish the null from the alternative hypothesis. It might seem strange that properties of the data themselves cannot be used to indicate how sensitive those data are. But *post hoc* or observed power, based on the effect size obtained in the data, gives no information about Type II error rate (Baguley, 2004): such observed power is determined by the *p*-value and thus gives no information in addition to that provided by the *p*-value, and thus gives no information about Type II error rate. (To see this, consider that a sensitive non-significant result would have a mean well below the minimally interesting mean; power calculated on the observed mean, i.e., observed power, would indicate the data insensitive, but it is the minimally interesting mean that should be used to determine power.) Intervals, such as confidence intervals solve the second problem; that is, they indicate how sensitive the data are, based on the very data themselves. But intervals do not solve the first problem, that of specifying minimally interesting values, as we shall see.

### INTERVAL ESTIMATES

**FIGURE 2:**
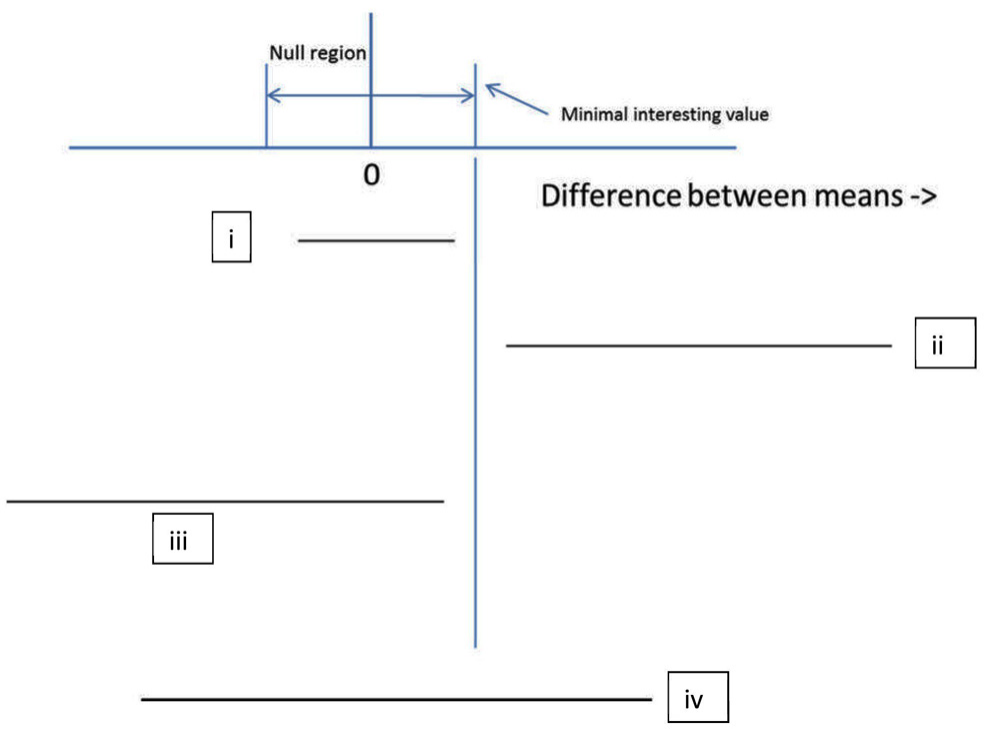
**The four principles of inference by intervals. (i)** If the interval is completely contained in the null region, decide that the population value lies in the null region (accept the null region hypothesis); **(ii)** If the interval is completely outside the null region, decide that the population value lies outside the null region (reject the null region hypothesis); **(iii)** If the upper limit of the interval is below the minimal interesting value, decide against a theory postulating a positive difference (reject a directional theory); **(iv)** If the interval includes both null region and theoretically interesting values, the data are insensitive (suspend judgment).

Interval estimates include confidence intervals, and their likelihood and Bayesian equivalents, namely, likelihood intervals and credibility intervals (also called highest density regions; see Dienes, 2008, for comparison). A confidence interval is the set of possible population values consistent with the data; all other population values may be rejected (see e.g., Smithson, 2003; Cumming, 2011). The APA recommends reporting confidence intervals whenever possible (American Psychological Association [APA], 2010). However, why should one report a confidence interval, unless it changes what conclusions might be drawn? Many statistics textbooks teach how to calculate confidence intervals but few teach how to use them to draw inferences about theory (for an exception see Lockhart, 1998). **Figure 2** shows the *four principles of inference by intervals* (adapted from Freedman and Spiegelhalter, 1983; Serlin and Lapsley, 1985; Rogers et al., 1993; Kruschke, 2010a; Berry et al., 2011). A non-significant result means that the confidence interval (of the difference, correlation, etc) contains the null value (here assumed to be 0). But if the confidence interval includes 0, it also includes some values either side of 0; so the data are always consistent with some population effect. Thus, in order to accept a null hypothesis, the null hypothesis must specify a null region, not a point value. Specifying the null region means specifying a minimally interesting value (just as for power), which forms either limit of the region. Then if the interval is contained within the null region, one can accept the null hypothesis: The only allowable population values are null values (rule i; cf equivalency testing, Rogers et al., 1993). If the interval does not cover the null region at all, the null hypothesis can be rejected. The only allowable population values are non-null (rule ii). Conversely if the interval covers both null and non-null regions, the data are insensitive: the data allow both null and non-null values (rule iv; see e.g., Kiyokawa et al., 2012 for an application of this rule).

In **Figure 1**, 95% confidence intervals are plotted. In all cases where the outcome was non-significant, the interval included not just 0 but also the true population effect (10). Given that 10 is agreed to be an interesting effect size, the confidence intervals show all the non-significant outcomes to be insensitive. In all those cases the data should not be used to count against the theory of a difference (rule iv). The conclusion follows without determining a scientifically relevant minimally interesting value. But a confidence interval can only be used to assert the null hypothesis when a null region can be specified (rule i; cf Hodges and Lehmann, 1954; Greenwald, 1975; Serlin and Lapsley, 1985; Cohen, 1988). For that assertion to be relevant to a given scientific context, the minimal value must be relevant to that scientific context (i.e., it cannot be determined by properties of the data alone nor can it be a generic default). For example, in clinical studies of depression it has been conventionally decided that a change of three units on the Hamilton scale is the minimal meaningful amount (e.g., Kirsch, 2010)^2^. Rule ii follows logically from rule i. Yet rule ii is stricter than current practice, because it requires the whole null region lie outside the interval, not just 0^3^.

While intervals can provide a systematic basis of inference which would advance current typical practice, they have a major problem in providing guidance in interpreting non-significant results. The problem is that asserting the null (and thus rejecting the null with a procedure that could have asserted it) requires in general specifying the minimally interesting value: What facts in the scientific domain indicate a certain value as a reasonable minimum? That may be the hardest part of a theory’s predictions to specify.^4^ If you find it hard to specify the minimal value, you have just run out of options for interpreting a non-significant result as far as orthodoxy is concerned.

### BAYES FACTORS

Bayes factors (*B*) indicate the relative strength of evidence for two theories (e.g., Berger and Delampady, 1987; Kass and Wasserman, 1996; Goodman, 1999; Lee and Wagenmakers, 2005; Gallistel, 2009; Rouder et al., 2009; Dienes, 2011; Kruschke, 2011).^5^ The Bayes factor *B* comparing an alternative hypothesis to the null hypothesis means that the data are *B* times more likely under the alternative than under the null. *B* varies between 0 and ∞, where 1 indicates the data do not favor either theory more than the other; values greater than 1 indicate increasing evidence for one theory over the other (e.g., the alternative over a null hypothesis) and values less than 1 the converse (e.g., increasing evidence for the null over the alternative hypothesis). Thus, Bayes factors allow three different types of conclusions: There is strong evidence for the alternative (*B* much greater than 1); there is strong evidence for the null (*B* close to 0); and the evidence is insensitive (*B* close to 1). This is already much more than *p*-values could give us.

In order to draw these conclusions we need to know how far from 1 counts as strong or substantial evidence and how close to 1 counts as the data being insensitive. Jeffreys et al. (1939/1961) suggested conventional cut-offs: A Bayes factor greater than 3 or else less than 1/3 represents substantial evidence; conversely, anything between 1/3 and 3 is only weak or “anecdotal” evidence.^6^ Are these just numbers pulled out of the air? In the examples considered below, when the obtained effect size is roughly that expected, a *p* of 0.05 roughly corresponds to a *B* of 3. This is not a necessary conclusion, nothing guarantees it; but it may be no coincidence that Jeffreys developed his ideas in Cambridge just as Fisher (1935) was developing his methods. That is, Jeffreys choice of three is fortunate in that it roughly corresponds to the standards of evidence that scientists have been used to using in rejecting the null. By symmetry, we automatically get a standard for assessing substantial evidence for the null: If 3 is substantial evidence for the alternative, 1/3 is substantial evidence for the null. (Bear in mind that there is no one-to-one correspondence of *B* with *p*-values, it depends on both the obtained effect size and also how precise or vague the predictions of the alternative are: With a sufficiently vague alternative a *B* of three may match a *p*-value of, for example, 0.01, or lower; Wetzels et al., 2011).

The Bayes factor is based on the principle that evidence supports the theory that most strongly predicted it. That is, in order to know how much evidence supports a theory, we need to know what the theory predicts. For the null hypothesis this is easy. Traditionally, the null hypothesis predicts that a single population value (typically 0) is plausible and all other population values are ruled out. But what does our theory, providing the alternative hypothesis, predict? Specifying the predictions of the theory is the difficult part of calculating a Bayes factor. The examples below, drawn partly from published studies, will show some straightforward methods theoretical predictions can be specified in a way suitable for Bayes factors. The goal is to determine a plot of the plausibility of different population values given the theory in its scientific context. Indeed, whatever one’s views on Bayes, thinking about justifications for minimum, typical or maximum values of an effect size in a scientific context is a useful exercise. We have to consider at least some aspect of such a plot to evaluate non-significant results; the minimum plausible value has to be specified for using power or confidence (credibility or likelihood) intervals in order to evaluate theories. That is, we should have been specifying theoretically relevant effect sizes anyway. We could get away without doing so, because specified effect sizes are not needed for calculating *p*-values; but the price has been incoherence in interpreting non-significant (as well as significant) results. It might be objected that having to specify the minimum was one of the disadvantages of inference by intervals; but as we will see in concrete examples, Bayes is more flexible about what is sufficient to be specified (e.g., rough expected value, or maximum), and different scientific problems end up naturally specifying predictions in different ways.

**FIGURE 3:**
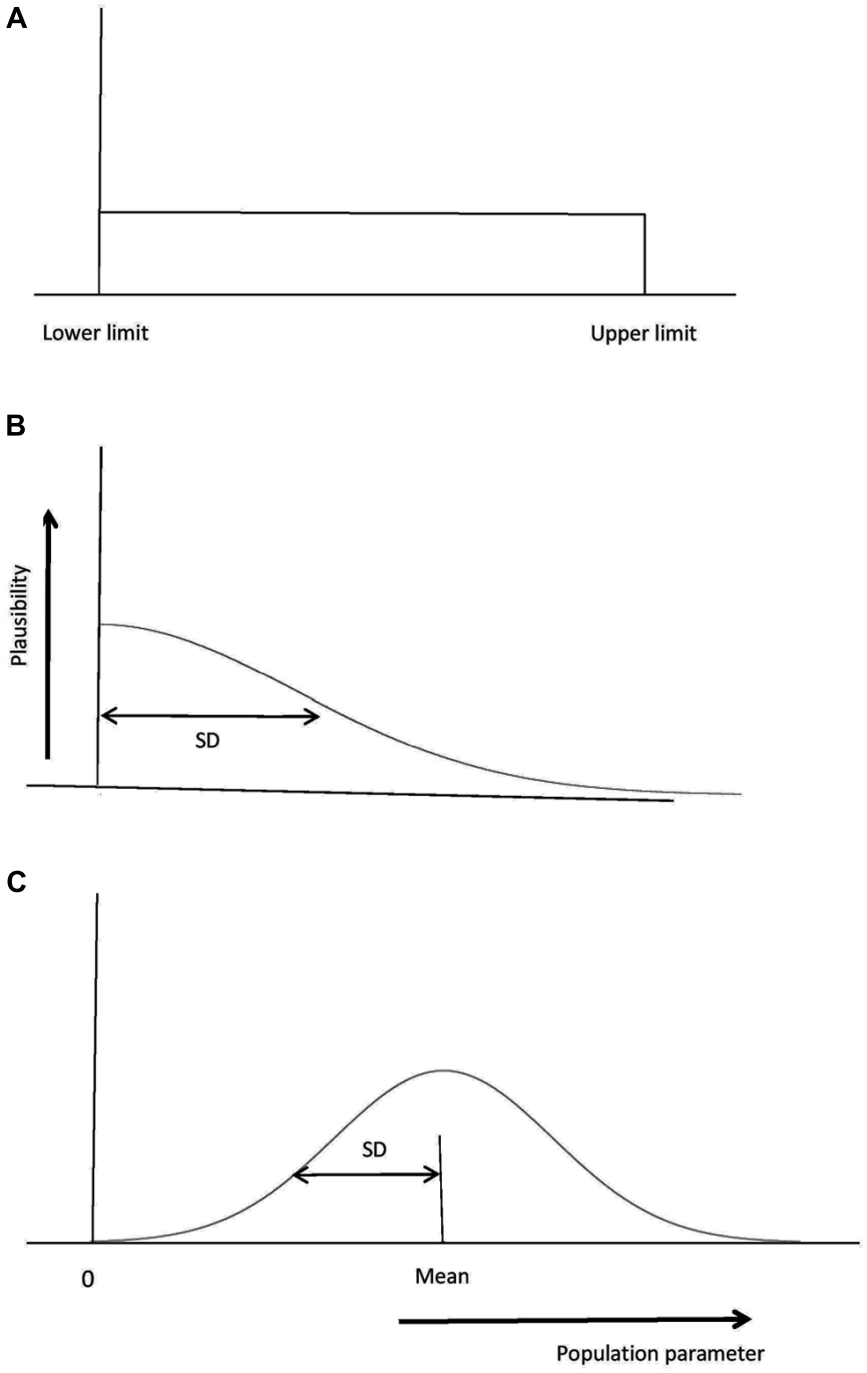
**Representing the alternative hypothesis. (A)** A uniform distribution with all population parameter values from the lower to the upper limit equally plausible. Here the lower limit is 0, a typical but not required value. **(B)** A normal distribution, with population parameter values close to the mean being more plausible than others. The SD also needs to be specified; a default of mean/2 is often useful. **(C)** A half-normal distribution. Values close to 0 are most plausible; a useful default for the SD is a typical estimated effect size. Population values less than 0 are ruled out.

For the free online Bayes factor calculator associated with Dienes (2008; http://www.lifesci.sussex.ac.uk/home/Zoltan_Dienes/inference/bayes_factor.swf), there are three distributions that can be used to represent the predictions of the theory: Uniform, normal or half-normal (see **Figure 3**). The properties of each distribution will be briefly described in turn, before considering concrete examples of Bayes factors in practice in the next section. Note these distributions plot the plausibility of different values of the population parameter being tested, for example the plausibility of population means or mean differences. The distributions in **Figure 3** in no way reflect the distribution of individual values in the population. The Dienes (2008) Bayes calculator assumes that the sampling distribution of the parameter estimate is normally distributed (just as a *t*-test does), and this obtains when, for example, the individual observations in the population are normally distributed. Thus, there may be a substantial proportion of people with a score below 0 in the population, and we could correctly believe this to be true while also assigning no plausibility to the population mean being below 0 (e.g., using a uniform from 0 to 10 in **Figure 3**). That is, using a uniform or half-normal distribution to represent the alternative hypothesis is entirely consistent with the data themselves being normally distributed. The distributions in **Figure 3** are about the plausibility of different population parameter values (e.g., population means of a condition or population mean differences or associations, etc.).

First, we consider the uniform. A researcher tests whether imagining a sporting move (e.g., a golf swing) for 15 min a day for a week improves measured performance on the move. She performs a *t*-test and obtains a non-significant result. Does that indicate that imagination in this context is not useful for improving the skill? That depends on what size effect is predicted. Just knowing that the effect is non-significant is in itself meaningless. Presumably, whatever size the population effect is, the imagination manipulation is unlikely to produce an effect larger than that produced by actually practicing the move for 15 min a day for a week. If the researcher trained people with real performance to estimate that effect, it could be used as the upper limit of a uniform in predicting performance for imagination. If a clear minimum level of performance can be specified in a way simple to justify, that could be used as the lower limit of the uniform; otherwise, the lower limit can be set to 0 (in practice, the results are often indistinguishable whether 0 or a realistic minimum is used, though in every particular case this needs to be checked; cf. Shang et al., 2013). In general, when another condition or constraint within the measurement itself determines an upper limit, the uniform is useful, with a default of 0 as the lower limit. An example of a constraint produced by the measurement itself is a scale with an upper limit; for a 0–7 liking scale, the difference between conditions cannot be more than seven. (The latter constraint is quite vague and we can usually do better; for example, by constraints provided by other data. See Dienes, in press, for examples of useful constraints created by types of scales.)

Next, we consider the normal distribution. Sometimes what is easily available is not another condition defining an upper limit, but data indicating a roughly likely value for an effect, should it exist. For example, it may be known for an implicit learning paradigm that asking people to search for rules rather than passively memorizing training material reduces classification accuracy in a later test phase by about 5%. A researcher may wish to change the manipulation by having people search for rules or not, not during the training phase as before, but between training and testing (cf. Mealor and Dienes, 2012). In the new experiment we may think 5% a reasonable estimate of the effect size that would be obtained (especially as we think similar mechanisms would be involved in producing the two effects). Further, if there is an effect in the post-training manipulation, we may have no strong reason to think it would be more than or less than the effect in the training manipulation. A natural way of representing this prediction of the theory is a normal distribution with a mean of 5%. What should the standard deviation (SD) of the normal distribution be? The theory predicts an effect in a certain direction; namely searching for rules will be harmful for performance. Thus, we require the plausibility of negative differences to be negligible. The height of a normal distribution comes pretty close to 0 by about two SDs out. Thus, we could set two SDs to be equal to the mean; that is, SD = mean/2. This representation uses another data set to provide a most likely value – but otherwise keeps predictions as vague as possible (any larger SD would involve non-negligible predictions in the wrong direction; any smaller SD would indicate more precision in our prediction). Using SD = mean/2 is a useful default to consider in the absence of other considerations; we will consider exceptions below. With this default, the effect is predicted to lie between 0 and twice the estimated likely effect size. Thus, we are in no way committed to the likely effect size; it just sets a ball park estimate, with the true effect being possibly close to the null value or indeed twice the likely value.

When there is a likely predicted effect size, as in the preceding example, there is another way we can proceed. We can use the half-normal distribution, with a mode at 0, and scale the half-normal distribution’s rate of drop by setting the SD equal to the likely predicted value. So, to continue with the preceding example, the SD would be set to 5%. The half-normal distribution indicates that smaller effect sizes are more likely than larger ones, and whatever the true population effect is, it plausibly lies somewhere between 0 and 10%. Why should one use the half-normal rather than the normal distribution? In fact, Bayes factors calculated either way are typically very similar; but the half-normal distribution considers values close to the null most plausible, and this often makes it hard to distinguish the alternative from the null. Thus, if the Bayes factor does clearly distinguish the theories with a half-normal distribution, we have achieved clear conclusions despite our assumptions. The conclusion is thereby strengthened. For this reason, a useful default for when one has a likely expected value is to use the half-normal distribution. (We will consider examples below to illustrate when a normal distribution is the most natural representation of a particular theory.)

To summarize, to know how much evidence supports a theory we need to know what the theory predicts. We have considered three possible ways of representing the predictions of the alternative, namely, the uniform, normal, and half-normal distributions. Representing the predictions of the alternative hypothesis is the part of performing Bayes that requires most thought. This thinking about the predictions of scientific theory can apparently be avoided by conventionally deciding on a default representation of the predictions of the alternative to use in all or most cases (e.g., Jeffreys et al., 1939/1961; Box and Tiao, 1973; Liang et al., 2008; Rouder et al., 2009; Rouder, 2012; Rouder and Morey, 2011; Wetzels and Wagenmakers, 2012; Wetzels et al., 2012). But as there is no default theory in science, a researcher still has the responsibility of determining whether the default representation used in any Bayes factor reasonably matches the predictions of their theory – and if it does not, that particular Bayes factor is not relevant for assessing the theory; one or more others will be. (See Vanpaemel and Lee, 2012, for the corresponding argument in computational modeling, that distributions over parameters are also best informed by scientific knowledge rather than general defaults.) Thus, Rouder et al. (2009, p. 232) recommend using content-specific constraints on specifying the alternative in the Bayes factor where this is possible. Vanpaemel (2010) also urges representing the alternative in a way as sensitive to theory as possible. Fortunately, more than a dozen papers have been published over the last couple of years illustrating the use of simple objectively-specified content-specific (non-default) constraints for Bayes factors in their particular scientific domains (e.g., Gallistel, 2009; Morey and Rouder, 2011; Newell and Rakow, 2011; Buhle et al., 2012; Dienes et al., 2012; Armstrong and Dienes, 2013; Dienes and Hutton, 2013; Fu et al., 2013a; Guo et al., 2013a,b; Mealor and Dienes, 2013a,b; Parris and Dienes, 2013; Semmens-Wheeler et al., 2013; Shang et al., 2013; Terhune et al., 2013). Some principles are illustrated below. The three possible ways of representing the alternative hypothesis considered here turn out to be sufficient for representing theoretical intuitions in many scientific contexts. While the alternative requires some thought, a default is used for the null in the Dienes (2008) calculator; it assumes the null hypothesis is that the true population value is exactly 0. We will consider in Appendix 1 how to change this according to scientific context as well (cf. Morey and Rouder, 2011, who also allow for flexible non-default nulls).

To know how much evidence supports a theory, we need to know what the evidence is. For a situation where a *t*-test or a *z*-test can be performed, the relevant summary of the data is the parameter estimate and its SE: For example, the sample mean difference and the SE of the difference. This is the same as for a *t*-test (or *z*-test); a *t* value just is the parameter estimate divided by its SE. So if one knows the sample mean difference, the relevant SE can be obtained by: mean difference/t, where t can be obtained through SPSS, R, etc. This formula works for a between-participants, within-participants, mixed or one sample *t*-test. Thus, any researcher reading a paper involving a *t*-test (or any *F* with one degree of freedom; the corresponding t is the square root of the *F*), or performing their own *t*-test (or *F* with one degree of freedom) can readily obtain the two numbers summarizing the data needed for the Bayes factor.

The Dienes (2008) calculator asks for the mean (i.e., parameter estimate in general, including mean difference) and the SE of this estimate. It also asks if the p(population value| theory; i.e., the plausibility of different population values given the theory) is uniform: If the answer is “yes,” boxes appear to enter the lower and upper bounds; if the answer is “no,” boxes appear to set the parameters of the normal distribution. In the latter case, to answer the question “Is the distribution one-tailed or two-tailed?” enter “1” for a half-normal and “2” for a normal.^7^

We can now use the simulations in **Figure 1** to illustrate some features of Bayes factors. **Table 1** shows the sequence of *p*-values obtained in the successive replications of the same experiment with a true population raw effect size of 10 units. Let us assume, as we did for interpreting the confidence intervals, that a value of 10 units is regarded as the sort of effect size that would hold if the theory were true. Thus, we can represent the alternative as a half-normal distribution with an SD of 10. **Table 1** shows both the “dance of the *p*-values” and the more graceful “tai chi of the Bayes factors.”

**Table 1:** Bayes factors corresponding to the *p*-values shown in **Figure 1**.

**(A)**
***p***	0.081	0.034*	0.74	0.034*	0.09	0.817	0.028*	0.001*	0.056	0.031*	0.279	0.024*	0.083
**B, giving support for:**													
Null													
Neither	2.96		0.52		2.70	0.46					1.73		2.96
Alternative		4.88		4.88			4.40	1024.6	3.33	4.88		4.28	
**(B)**
***p***	0.002*	0.167	0.172	0.387	0.614	0.476	0.006*	0.028*	0.002*	0.024*	0.144	0.23	
**B, giving support for:**													
Null													
Neither		2.16	2.12	1.01	0.65	0.75					2.36	1.73	
Alternative	49.86						28.00	4.28	49.86	5.60			

The *B*s are sorted into three categories: The top row is for *B*s less than 1/3 (support for null), the bottom row for *B*s more than 3 (support for alternative), and the middle row is for *B*s in the middle, indicating data insensitivity (support for neither hypothesis). Significant *p*-value’s are asterisked.

In interpreting **Table 1**, remember that a *B* above 3 indicates substantial support for the alternative and a *B* less than 0.33 indicates substantial support for the null. In **Table 1**, significant results are associated with *B*s above 3 (because, as it happens, the effect sizes in those cases are around the values we regarded as typical, and not close to 0; in fact for a fixed *p* = 0.05, *B* will indicate increasing support for the null as *N* increases, and thus the sample mean shrinks; Lindley, 1957). Note also that Bayes factors can be quite large (e.g., 1024); they do not scale according to our intuitions trained on *t*-values^8^.

Crucially, in **Table 1**, non-significant results correspond to Bayes factors indicating insensitivity, that is, between 3 and 1/3. (It is in no way guaranteed of course that Bayes factors won’t sometimes indicate evidence for the null when the null is false. But this example illustrates how such misleading Bayes factors would be fewer than non-significant *p*-values when the null is false. For analytically determined general properties of error rates with Bayes factors contrasting simple hypotheses see Royall, 1997.) A researcher obtaining any of the non-significant results in **Table 1**, would, following a Bayesian analysis, conclude that the data were simply insensitive and more participants should be run. The same conclusions follow from using confidence intervals, as shown in **Figure 1**, using the rules of inference in **Figure 2**. So, if Bayes factors often produce answers consistent with inference by intervals, what does a Bayes factor buy us? It will allow us to assert the null without knowing a minimal interesting effect size, as we now explore. We will see that a non-significant result sometimes just indicates insensitivity, but it is sometimes support for the null.

## EXAMPLES USING THE DIFFERENT WAYS OF REPRESENTING THE PREDICTIONS OF THE THEORY

We will consider the uniform, normal, and half-normal in turn. The sequence of examples serve as a tutorial in various aspects of calculating Bayes factors and so are best read in sequence.

### BAYES WITH A UNIFORM DISTRIBUTION

#### A manipulation is predicted to decrease an effect

Dienes et al. (2012) predicted that in a certain context a negative mood would reduce a certain type of learning. To simplify so as to draw out certain key points, the example will depart from the actual paradigm and results of Dienes et al. (2012). Subjects perform a two-alternative forced choice measure of learning, where 50% is chance. If performance in the neutral condition is 70%, then, if the theory is correct, performance in the negative mood condition will be somewhere between 50 and 70%. That is, the effect of mood would be somewhere between 0% and 20%. Thus, the predictions of a theory of a mood effect could be represented as a uniform from 0 to 20 (in fact, there is uncertainty in this estimate which could be represented, but we will discuss that issue in the subsequent example).

Performance in the negative mood condition is (say) 65% and non-significantly different from that in the neutral condition, *t*(50) = 0.5, *p* = 0.62. So the mean difference is 70% – 65% = 5%. The SE = (mean difference)/*t* = 10%. Entering these values in the calculator (mean = 5, SE= 10, uniform from 0 to 20) gives *B* = 0.89. That is, the data are insensitive and do not count against the theory that predicted negative mood would impair performance (indeed, Dienes et al. (2012) obtained a non-significant result which a Bayes factor indicated was insensitive).

If the performance in the negative mood condition had been 70%, the mean difference between mood conditions would be 0. The Bayes factor is then 0.60, still indicating data insensitivity. That is, obtaining identical sample means in two conditions in no way guarantees that there is compelling evidence for the null hypothesis. If the performance in the negative mood condition had been 75%, the mean difference between mood conditions would be 5% in the wrong direction. This is entered into the calculator as –5 (i.e., as a negative number: The calculator assumes positive means are in the direction predicted by theory if the theory is directional). *B* is then 0.43, still insensitive. That is, having the means go in the wrong direction does not in itself indicate compelling evidence against the theory (even coupled with a “very non-significant” *p* of 0.62). If the SE were smaller, say 5 instead of 10, then a difference of –5 gives a *B* of 0.16, which is strong evidence for the null and against the theory. That is, as the SE shrinks, the data become more sensitive, as would be expected.

The relation between SE and sensitivity allows an interesting contrast between *B* and *p*-values. *p*-values do not monotonically vary with evidence for the null: A larger *p*-value may correspond to less evidence for the null. Consider a difference of 1 and a SE of 10. This gives a *t* of 0.01, *p* = 0.99. If the difference were the same but the SE were much smaller, e.g., 1, *t* would be larger, 1 and *p* = 0.32. The *p*-value is smaller because the SE is smaller. To calculate *B*, assume a uniform from 0 to 20, as before. In the first case, where *p* = 0.99, *B* is 0.64 and in the second case, where *p* = 0.32, *B* = 0.17. *B* is more sensitive (and more strongly supports the null) in the second case precisely because the SE is lower in the second case. Thus, a high *p*-value may just indicate the SE is large and the data insensitive. It is a fallacy to say the data are convincing evidence of the null just because the *p*-value is very high. The high *p*-value may be indicating just the opposite.

#### Testing additivity vs interaction

Buhle et al. (2012) wished to test whether placebo pain relief works through the same mechanism as distraction based pain relief. They argued that if it were separate mechanisms, the effect of placebo should be identical in a distraction vs control condition; if the same mechanism, then placebo would have less effect in a distraction condition. Estimating from their **Figure 2**, the effect of placebo vs no placebo in the no distraction condition was 0.4 pain units (i.e., placebo reduced pain by 0.4 units). The effect of placebo in the distraction condition was 0.44 units (estimated). The placebo × distraction interaction raw effect (the difference between the two placebo effects) is therefore 0.4 – 0.44 = –0.04 units (note that it is in the wrong direction to the theory that distraction would reduce the placebo effect). The interaction was non-significant, *F*(1,31) = 0.109, *p* = 0.746. But in itself the non-significant result does not indicate evidence for additivity; perhaps the data were just insensitive. How can a Bayes factor be calculated?

The predicted size of the interaction needs to be specified. Following Gallistel (2009), Buhle et al. (2012) reasoned that the maximum size of the interaction effect would occur if distraction completely removed the placebo effect (i.e., placebo effect with distraction = 0). Then, the interaction would be the same size as the placebo effect in the no distraction condition (that is, interaction = placebo effect with no distraction – placebo effect with distraction (i.e., 0.4 – 0) = placebo effect with no distraction = 0.4). The smallest the population interaction could be (on the theory that distraction reduces the placebo effect) is if the placebo effect was very nearly the same in both conditions, that is an interaction effect of as close as we like to 0. So we can represent the plausible sizes of the interaction as a uniform from 0 to the placebo effect in the no distraction condition, that is from 0 to 0.4.

The *F* of 0.109 corresponds to a *t*-value of $\sqrt{0.109}$ = 0.33. Thus the SE of the interaction is (raw interaction effect)/*t* = 0.04/0.33 = 0.12. With these values (mean = 0.04, SE = 0.12, uniform from 0 to 0.4), *B* is 0.29, that is, substantial evidence for additivity. This conclusion depends on assuming that the maximum the interaction could be was the study’s estimate of the placebo effect with no distraction. But this is just an estimate. We could represent our uncertainty in that estimate – and that would always push the upper limit upward. The higher the upper limit of a uniform, the vaguer the theory is. The vaguer the theory is, the more Bayes punishes the theory, and thus the easier it is to get evidence for the null. As we already have evidence for the null, representing uncertainty will not change the qualitative conclusion, only make it stronger. The next example will consider the rhetorical status of this issue in the converse case, when the Bayes factor is insensitive.

In sum, no more data need to be collected; the data are already sensitive enough to make the point. The non-significant result was rightly published, given it provided as substantial evidence for the null as a significant result might against it.

#### Interactions that can go in both directions

In the above example, the raw interaction effect was predicted on theory to go in only one direction, that is, to vary from 0 up to some positive maximum. We now consider a similar example, and then generalize to a theory that allows positive and negative interactions. The latter sort of interaction may be more difficult to specify limits in a simple way, but in this example we can.

Watson et al. (2003) compared the effectiveness of Process Experiential Therapy (PET; i.e., Emotion Focussed Therapy) with Cognitive Behavioral Therapy (CBT) in treating depression. There were a variety of measures, but for illustration we will look at just the Beck Depression Inventory (BDI) on the entire intent-to-treat sample (*n* = 93). CBT reduced the BDI from pre- to post-test (from 25.09 to 12.56), a raw simple effect of time of 12.53. Similarly, PET reduced the BDI from 24.50 to 13.05, a raw simple effect of 11.45. The *F* for the group (CBT vs PET) × time (pre vs post) interaction was 0.18, non-significant. In itself, the non-significant interaction does not mean the treatments are equivalent in effectiveness. To know what it does mean, we need to get a handle on what size interaction effect we would expect.

One theory is based on assuming we know that CBT does work and is bound to be better than any other therapy for depression. Then, the population interaction effect (effect of time for CBT – effect of time for PET) will vary between near 0 (when the effects are near equal) and the effect of time for CBT (when the other therapy has no effect). Thus assuming 12.53 to be a sensitive estimate of the population effect of CBT, we can use a uniform for the alternative from 0 to 13 (rounding up). The sample raw interaction effect is 12.53-11.45 = 1.08. The *F* for the interaction (0.18) corresponds to a *t* of $\sqrt{0.18}$ = 0.42. Thus the SE of the raw interaction effect is 1.08/0.42 = 2.55. With these values for the calculator (mean = 1.08, SE = 2.55, uniform from 0 to 13), *B* = 0.36.

The *B* just falls short of a conventional criterion for substantial evidence for equivalence. The evidence is still reasonable, in that in Bayes sharp conventional cut offs have no absolute meaning (unlike orthodoxy); evidence is continuous^9^. Further, we assumed we had estimated the population effect of CBT precisely. We could represent some uncertainty in that estimate, e.g., use an upper limit of a credibility or confidence interval of the effect of CBT as the upper limit of our uniform. But we might decide as a defeasible default not to take into account uncertainty in estimates of upper limits of uniforms when we have non-significant results. In this way, more non-significant studies come out as inconclusive; that is, we have a tough standard of evidence for the null.

It is also worth considering another alternative hypothesis, namely one which asserts that CBT might be better than PET – or *vice versa*. We can represent the predictions of this hypothesis as a uniform from -11 to +13. (The same logic is used for the lower limit; i.e., if PET is superior, the most negative the interaction could be is when the effect of CBT is negligible compared to PET, so interaction = -effect of time for PET.) Then *B* = 0.29. That is, without a pre-existing bias for CBT, there is substantial evidence for equivalence in the context of this particular study (and it would get even stronger if we adjusted the limits of the uniform to take into account uncertainty in their estimation). Even with a bias for thinking CBT is at least the best, there is modest evidence for equivalence.

#### Interaction with degrees of freedom more than 1 and simple effects

Raz et al. (2005) have demonstrated a remarkable effect of hypnotic suggestion in a series of studies: Suggesting that the subject cannot read words, that the stimuli will appear as a meaningless script, substantially reduces the Stroop effect. **Table 2** presents imaginary but representative data.

**Table 2:** (Imaginary) means for the effectiveness of a hypnotic suggestion to reduce the Stroop effect.

	Incongruent	Neutral	Congruent
No Suggestion	850	750	720
Suggestion	785	745	715

The crucial test of the hypothesis that the suggestion will reduce the Stroop effect is the Suggestion (present vs absent) × Word Type (incongruent vs neutral vs congruent) interaction. This is a 2-degree of freedom effect. However, it naturally decomposes into two 1-degree of freedom effects. The Stroop effect can be thought of as having two components with possibly different underlying mechanisms: An interference effect (incongruent - neutral) and a facilitation effect (neutral - congruent). Thus the interaction decomposes into the effect of suggestion on interference and the effect of suggestion on facilitation. In general, multi-degree of action effects often decompose into one degree of freedom contrasts addressing specific theoretic questions. Let’s consider the effect of suggestion on interference (the same principles of course apply to the effect of suggestion on facilitation). First, let us say the test for the interaction suggestion (present vs absent) × word type (incongruent vs neutral) is *F*(1,40) = 2.25, *p* = 0.14. Does this mean the effect of suggestion is ineffective in the context of this study for reducing interference?

The *F* of 2.25 corresponds to a *t* of $\sqrt{2.25}$ = 1.5. The raw interaction effect is (interference effect for no suggestion) - (interference effect for suggestion) = (850 - 750) - (785 - 745) = 100 - 40 = 60. Thus, the SE for the interaction is 60/1.5 = 40. What range of effects could the population effect plausibly be? If the suggestion had removed the interference effect completely, the interaction would be the interference effect for no suggestion (100); conversely if the suggestion had been completely ineffective, it would leave the interference effect as it is, and thus the interaction would be 0. Thus, we can represent the alternative as a uniform from 0 to 100. This gives a *B* of 2.39. That is, the data are insensitive but if anything should increase one’s confidence in the effectiveness of the suggestion.

Now, let us say the test of the interaction gave us a significant result, e.g., *F*(1,40) = 4.41, *p* = 0.04, but the means remain the same as in **Table 2**. Now the F corresponds to a t of $\sqrt{4.41}$ = 2.1. The raw size of interaction is still 60; thus the SE is 60/2.1 = 29. *B* is now 5.54, substantial evidence for the effectiveness of suggestion. In fact, the test of the interference effect with no suggestion is significant, *t*(40) = 2.80, *p* = 0.008; and the test for the interference effect for just the suggestion condition gives *t*(40) = 1.30, *p* = 0.20. Has the suggestion eradicated the Stroop interferenc effect?

We do not know if the Stroop effect has been eradicated or just reduced on the basis of the last non-significant result. A Bayes factor can be used to find out. The interference effect is 40. So the SE is 40/1.3 = 31. On the alternative that the interference effect was not eradicated, the effect will vary between 0 and the effect found in the no suggestion condition; that is, we can use a uniform from 0 to 100. This gives a *B* of 1.56. That is, the data are insensitive, and while we can conclude that the interference effect was reduced (the interaction indicates that), we cannot yet conclude whether or not it was abolished.

#### Paired comparisons

Tan et al. (2009, 2014) tested people on their ability to use a Brain–Computer Interface (BCI). People were randomly assigned to three groups: no treatment, 12 weeks of mindfulness meditation, and an active control (12 weeks of learning to play the guitar). The active control was designed (and shown) to create the same level of expectations as mindfulness meditation in improving BCI performance. Initially, eight subjects were run in each group. Pre-post differences on BCI performance were -8, 15, and 9 for the no-treatment, mindfulness, and active control, respectively. The difference between mindfulness and active control was not significant, *t*(14) = 0.61, *p* = 0.55. So, is mindfulness any better than an active control?

Assume the active control and mindfulness were equalized on non-specific processes like expectations and beliefs about change, and mindfulness may or may not contain an additional active component. Then, the largest the difference between mindfulness and active control could be is the difference between mindfulness and the no-treatment control (i.e., a difference of 23, which was significant). Thus, the alternative can be represented a uniform from 0 to 23. The actual difference was 15 - 9 = 6, with a SE of 6/0.61 = 9.8. This gives a *B* of 0.89. The data were insensitive. (In fact, with degrees of freedom less than 30, the SE should be increased slightly by a correction factor given below. Increasing the SE reduces sensitivity.)

Thus, the following year another group of participants were run. One cannot simply top up participants with orthodox statistics, unless pre-specified as possible by one’s stopping rule (Armitage et al., 1969); by contrast, with Bayes, one can always collect more participants until the data are sensitive enough, that is, *B* < 1/3 or *B* > 3; see e.g., Berger and Wolpert (1988), Dienes (2008, 2011). Of course, *B* is susceptible to random fluctuations up and down; why cannot one capitalize on these and stop when the fluctuations favor a desired result? For example, Sanborn and Hills (2014) and Yu et al. (2014) show that if the null is true, stopping when *B* > 3 (if that ever occurs) increases the proportion of cases that *B* > 3 when the null is true. However, as Rouder (2014) shows, it also increases the proportion of cases that *B* > 3 when Ho is false, and to exactly the same extent: *B* retains its meaning as relative strength of evidence, regardless of stopping rule (for more discussion, see Dienes, forthcoming).

With all data together (*N* = 63), the means for the three groups were 2, 14, and 6, for no-treatment, mindfulness and active control, respectively. By conventional statistics, the difference between the mindfulness group and either of the others was significant; for mindfulness vs meditation, *t*(41) = 2.45, *p* = 0.019. The interpretation of the latter result is compromised by the topping up procedure (one could argue the *p* is less than 0.05/2, so legitimately significant at the 5% level; however, let us say the result had still been insensitive, would the researchers have collected more data the following year? If so, the correction should be more like 0.05/3; see Strube, 2006; Sagarin et al., 2014). A Bayes factor indicates strength of evidence independent of stopping rule, however. Assume as before that the maximum the plausible difference between mindfulness and active control could be is the difference between mindfulness and no-treatment, that is, 12. We represent the alternative as a uniform from 0 to 12. The mean difference between mindfulness and active control is 14 - 6 = 8, with a SE of 8/2.45 = 3.3. This gives a *B* of 11.45, strong evidence for the advantage of mindfulness over an active control.

### BAYES WITH A HALF-NORMAL DISTRIBUTION

In the previous examples we considered cases where a rough plausible maximum could be determined. Here, we consider an illustrative case where a rough expected value can be determined (and the theory makes a clear directional prediction).

#### Norming materials

Guo et al. (2013b) constructed lists of nouns that differed in terms of the size of the object that the words represented, as rated on a 1–7 bipolar scale (1 = very small; 7 = very big). Big objects were perceived as bigger than small objects (5.42 vs 2.73), *t*(38) = 20.05, as was desired. Other dimensions were also rated including height. Large objects were rated non-significantly taller than small objects on an equivalent scale (5.13 vs 4.5), *t*(38) = 1.47, *p* = 0.15. Are the materials controlled for height? The difference in size was 5.42 - 2.73 = 2.69. This was taken to be a representative amount by which the two lists may differ on another dimension, like height. Thus, a *B* was calculated assuming a half-normal with SD = 2.69. (The test is directional because it is implausible that big objects would be shorter, unless specially selected to be so.) The “mean” was 5.13 - 4.5 = 0.63 height units, the SE = 0.63/1.47 = 0.43 height units. This yields *B* = 0.83. The data are insensitive; no conclusions follow as to whether height was controlled. (Using the same half-normal, the lists were found to be equivalent on familiarity and valence, *B*s < 1/3. These dimensions would be best analyzed with full normal distributions (i.e., mean of 0, SD = 2.69), but if the null is supported with half-normal distributions it is certainly supported with full normal distributions, because the latter entail vaguer alternatives.)

### BAYES WITH A NORMAL DISTRIBUTION

A half-normal distribution is intrinsically associated with a theory that makes directional predictions. A normal distribution may be used for theories that do not predict direction, as well as those that do. In the second case, the normal rather than half-normal is useful where it is clear that a certain effect is predicted and smaller effects are increasingly unlikely. One example is considered.

*Theory roughly predicts a certain value and smaller values are not more likely*. Fu et al. (2013b) tested Chinese and UK people on ability to implicitly learn global or local structures. A culture (Chinese vs British) by level (global vs local) interaction was obtained. Chinese were superior than British people at the global level, RT difference in learning effects = 50 ms, with SE= 14 ms. The difference at the local level was 15 ms, SE = 13 ms, *t*(47) = 1.31, *p* = 0.20. Fu et al. (2013b) wished to consider the theory that Chinese outperform British people simply because of greater motivation. If this were true, then Chinese people should outperform British people at the local level to the same extent as they outperform British at the global level. We can represent our knowledge of how well this is by a normal distribution with a mean of 50 and a SD of 14. This gives *B* = 0.25. That is, relative to this theory, the null is supported. The motivation theory can be rejected based both on the significant interaction and on the Bayes factor.

My typical default for a normal distribution is to use SD = mean/2. This would suggest an SD of 25 rather than 14, the latter being the SD of the sampling distribution of the estimated effect in the global condition. In this case, where the same participants are being run on the same paradigm with a minor change to which motivation should be blind, the sampling distribution of the effect in one condition stands as a good representation of our knowledge of its effect in the other condition, assuming a process blind to the differences between conditions^10^. Defaults are good to consider, but they are not binding.

## SOME GENERAL CONSIDERATIONS IN USING BAYES FACTORS

Appendix 1 gives further examples, illustrating trend analysis, the use of standardized effect sizes, and why sometimes raw effect sizes are required to address scientific problems; it also indicates how to easily extend the use of the Dienes (2008) Bayes calculator to simple meta-analysis, correlations, and contingency tables. Appendix 2 considers other methods for comparing relative evidence (e.g., likelihood inference and BIC, the Bayesian Information Criterion). Appendix 3 considers how to check the robustness of conclusions that follow from Bayes factors.

### ASSUMPTIONS OF THE DIENES (2008) CALCULATOR

The calculator assumes the parameter estimate is normally distributed with known variance. However, in a *t*-test situation we have only estimated the variance. If the population distribution of observations is normal, and degrees of freedom are above 30, then the assumption of known variance is good enough (see Wilcox, 2010, for problems when the underlying distribution is not normal). For degrees of freedom less than 30, the following correction should be applied to the SE: Increase the SE by a factor (1 + 20/df × df) (adapted from Berry, 1996; it produces a good approximation to *t*, over-correcting by a small amount). For example if the df = 13 and the SE of the difference is 5.1, we need to correct by [1 + 20/(13 × 13)] = 1.12. That is, the SE we will enter is 5.1 × 1.12 = 5.7. If degrees of freedom have been adjusted in a *t*-test (on the same comparison as a Bayes factor is to be calculated for) to account for unequal variances, the adjusted degrees of freedom should be used in the correction. No adjustment is made when the dependent variable is (Fisher *z* transformed) correlations or for the contingency table analyses illustrated in the Appendix, as in these cases the SE is known.

Note that the assumptions of the Dienes (2008) calculator are specific to that calculator and not to Bayes generally. Bayes is a general all-purpose method that can be applied to any specified distribution or to a bootstrapped distribution (e.g., Jackman, 2009; Kruschke, 2010a; Lee and Wagenmakers, 2014; see Kruschke, 2013b, for a Bayesian analysis that allows heavy-tailed distributions). Bayes is also not limited to one degree of freedom contrasts, as the Dienes (2008) calculator is (see Hoijtink et al., 2008, for Bayes factors on complex hypotheses involving a set of inequality constraints). However, pin point tests of theoretical predictions are generally one degree of freedom contrasts (e.g., Lewis, 1993; Rosenthal et al., 2000). Multiple degree of freedom tests usually serve as precursors to one degree of freedom tests simply to control familywise error rates (though see Hoijtink et al., 2008). But for a Bayesian analysis one should not correct for what other tests are conducted (only data relevant to a hypothesis should be considered to evaluate that hypothesis), so one can go directly to the theoretically relevant specific contrasts of interest (Dienes, 2011; for more extended discussion see Dienes, forthcoming; and Kruschke, 2010a for the use of hierarchical modeling for dealing with multiple comparisons).

### POWER, INTERVALS, AND BAYES

No matter how much power one has, a sensitive result is never guaranteed. Sensitivity can be guaranteed with intervals and Bayes factors: One can collect data until the interval is smaller than the null region and is either in or out of the null region, or until the Bayes factor is either greater than three or less than a third (see Dienes, 2008, in press for related discussion; in fact, error probabilities are controlled remarkably well with such methods, see Sanborn and Hills, 2014, for limits and exceptions, and Rouder, 2014, for the demonstration that the Bayes factor always retains its meaning as strength of evidence – how much more likely the data are on H1 than H0 – regardless of stopping rule). Because power does not make use of the actual data to assess its sensitivity, one should assess the actual sensitivity of obtained data with either intervals or Bayes factors. Thus, one can run until sensitivity has been reached. Even if one cannot collect more data, there is still no point referring to power once the data are in. The study may be low powered, but the data actually sensitive; or the study may be high powered, and the data actually insensitive. Power is helpful in finding out a rough number of observations needed; intervals and Bayes factors can be used thereafter to assess data (cf. Royall, 1997).

Consider a study investigating whether imagining kicking a football for 20 min every day for a month increased the number of shots into goal out of 100 kicks. Real practice in kicking 20 min a day increases performance by 12 goals. We set this as the maximum of a uniform. What should the minimum be? The minimum is needed to interpret a confidence or other interval. It is hard to say for sure what the minimum should be. A score of 0.01 goals may not be worth it, but maybe 1 goal would be? Let us set 0.5 as the minimum.

The imagination condition led to an improvement of 0.4 goals with a SE of 1. Using a uniform from 0.5 to 12, *B* = 0.11, indicating substantial evidence for the null hypothesis. If we used a uniform from 0 to 12, *B* = 0.15, hardly changed. However, a confidence (or other) interval would declare the results insensitive, as the interval extends outside the null region (even a 68% interval, provided by 0.4 ± 1 goals). The Bayes factor makes use of the full range of predictions of the alternative hypothesis, and can use this information to most sensitively draw conclusions (conditional on the information it assumes; Jaynes, 2003). Further, the Bayes factor is most sensitive to the maximum, which could be specified reasonably objectively. Inference by intervals is completely dependent on specification of the minimum, which is often hard to specify objectively. However, if the minimum were easy to objectively specify and the maximum hard in a particular case, it is inference by intervals that would be most sensitive to the known constraints, and would be the preferred solution. In that sense, Bayes factors and intervals complement each other in their strengths and weaknesses (see **Table 3**)^11^.

**Table 3:** Comparing intervals and Bayes for interpreting a non-significant result.

	What does it tell you?	What do you need to link data to theory?	Amount of data needed to obtain evidence for the null?	What would be a useful stopping rule to guarantee sensitivity?

Intervals	How precisely a parameter has been estimated; a reflection of data rather than theory.	A minimal value below which the theory is refuted.	Enough to make sure the width of the interval is less than that of the null region; considerable participant numbers will typically be needed in contrast to Bayes factors.	Interval width no more than null region width and interval either completely in or completely out of the null region.
Bayes factors	The strength of evidence the data provide for one theory over another; specific to the two theories contrasted.	A rough expected value or maximum value consistent with theory.	Bayes factors ensure maximum efficiency in use of participants, given a Bayes factor measures strength of evidence.	Bayes factor either greater than three or less than a third.

In sum, in many cases, Bayes factors may be useful not only when there is little chance of collecting more data, so as to extract the most out of the data; but also in setting stopping rules for grant applications, or schemes like Registered Reports in the journal Cortex, so as to collect data in the most efficient way (for an example see Wagenmakers et al., 2012). (By estimating the relevant SD and likely population effect in advance of running an experiment, you can enter into the calculator different SEs based on different *N*, and find the *N* for which *B* > 3 or <1/3. This provides an estimate of the required N. But, unlike with orthodox statistics, it is not an estimate you have committed to; cf. Royall, 1997; Kruschke, 2010a).

### WHY CHOOSE ONE DISTRIBUTION RATHER THAN ANOTHER?

On a two-alternative recognition test we have determined that a rough likely level of performance, should knowledge exist, is 60%, and we are happy that it is not very plausible for performance to exceed 70%, nor to go below chance baseline (50%). Performance is 55% with a SE of 2.6%. To determine a Bayes factor, we need first to rescale so that the null hypothesis is 0. So we subtract 50% from all scores. Thus, the “mean” is 5% and the SE 2.6%. We can use a uniform from 0 to 20 to represent the constraint that the score lies between chance and 20% above chance. This gives *B* = 2.01.

But we might have argued that as 60% (i.e., 10% above baseline) was rather likely on the theory (and background knowledge) we could use a normal distribution with a mean of 10% and a SD of mean/2 = 5%. (Note this representation still entails that the true population value is somewhere between roughly 0 and 20.) This gives *B* = 1.98, barely changed. Or why not, you ask, use a half-normal distribution with the expected typical value, 10%, as the SD? (Note this representation still entails that the true population value is somewhere between 0 and roughly 20.) This gives *B* = 2.75, somewhat different but qualitatively the same conclusion. In all cases the conclusion is that more evidence is needed. (Compare also the different ways of specifying the alternative provided by Rouder et al., 2009; see Appendix 3 for comparisons.) In this case, there may be no clear theoretical reason for preferring one of the distributions over the other for representing the alternative. But as radically shifting the distribution around (from flat to humped right up against one extreme to humped in the middle) made no qualitative difference to conclusions we can trust the conclusions.

Always perform a robustness check: Could theoretical constraints be just as readily represented in a different way, where the real theory is neutral to the different representations? Check that you get the same conclusion with the different representations. If not, run more participants until you do. If you cannot collect more data, acknowledge ambiguity in the conclusion (as was done by e.g., Shang et al., 2013). (See Kruschke, 2013c, for the equivalent robustness check for inference by intervals.) Appendix 3 considers robustness in more detail.

Different ways of representing the alternative may clearly correspond to different theories, as was illustrated in example 2 in Appendix 1. Indeed, one of the virtues of Bayes is that it asks one to consider theory, and the relation of theory to data, carefully.

### BUT HOW CAN ANY CONSTRAINTS BE SET ON WHAT MY ALTERNATIVE PREDICTS?

The examples in this paper (including the appendix) have illustrated finding constraints by use of data from different conditions, constraints intrinsic to the design and logic of the theory, and those provided by standardized effect sizes and regression slopes to convert different raw units of measurement into each other; and Dienes (in press) provides examples where constraints intrinsic to the measurement scale are very useful. However, there is one thing one cannot do, and that is to use the very effect itself, or its confidence interval, as the basis for predicting that same effect. For example, if the obtained mean difference was 15 ms, one cannot for that reason set the alternative to a half-normal distribution with a SD of 15. One can use other aspects of the same data to provide constraints, but not the very aspect that one is testing (Jaynes, 2003). Double counting the mean difference in both the data summary and as specifying the predictions of the alternative violates the axioms of probability in updating theory probabilities. This problem creates pressure to demand default alternatives for Bayes factors, on the grounds that letting people construct their own alternative is open to abuse (Kievit, 2011).

The Bayes factor stands as a good evaluation of a theory to the extent that its assumptions can be justified. And fortunately, all assumptions that go into a Bayes factor are public. So, the type of abuse peculiar to Bayes (that is, representing what a theory predicts in ways favorable with respect to how the data actually turned out) is entirely transparent. It is open to public scrutiny and debate (unlike many of the factors that affect significance testing; see Dienes, 2011). Specifying what predictions a theory makes is also part of the substance of science. It is precisely what we should be trying to engage with, in the context of public debate. Trying to convince people of one’s theory with cheap rhetorical tricks is the danger that comes with allowing argument over theories at all. Science already contains the solution to that problem, namely transparency, the right of everyone in principle to voice an argument, and commitment to norms of accountability to evidence and logic. When the theory itself does work in determining the representation of the alternative, the Bayes factor genuinely informs us about the bearing of evidence on theory.

### SUBJECTIVE VS OBJECTIVE BAYES

Bayesian approaches are often divided into subjective Bayes and objective Bayes (Stone, 2013). Being Bayesian at all in statistics is defined by allowing probabilities for population parameter values (and for theories more generally), and using such probability distributions for inference. Whenever one of the examples above asked for a specification what the theory predicts, it meant an assignment of a probability density distribution to different parameter values. How are these probabilities to be interpreted? The subjective Bayesian says that these probabilities can only be obtained by looking deep in one’s soul; probabilities are intrinsically subjective (e.g., Howson and Urbach, 2006). That is, it is all a matter of judgment. The objective Bayesian says that rather than relying on personal or subjective judgment, one should describe a problem situation such that the conclusions objectively follow from the stated constraints; every rational person should draw the same conclusions (e.g., Jaynes, 2003).

The approach in this paper respects both camps. The approach is objective in that the examples illustrate rules of thumb that can act as (contextually relevant) defaults, where the probability distributions are specified in simple objective ways by reference to data or logical or mathematical relations inherent in the design. No example relied on anyone saying, “according to my intuition the mean should be two because that’s how I feel” (cf. Howard et al., 2000, for such priors). But the approach is subjective in that the examples illustrate that only scientific judgment can determine the right representation of the theory’s predictions given the theory and existing background knowledge; and that scientific judgment entails that all defaults are defeasible – because science is subject to the frame problem, and doing Bayes is doing science. Being a subjectivist means a given Bayes factor can be treated not as THE objective representation of the relation of the theory to evidence, but as the best given the current level of discussion, and it could be improved if e.g., another condition was run which informed what the theory predicted in this context (e.g., defined a maximum value more clearly). A representation of the theory can be provisionally accepted as reflecting current judgment and ignorance about the theory.

## CONCLUSION

This paper has explored the interpretation of non-significant results. A key aspect of the argument is that non-significant results cannot be interpreted in a theoretical vacuum. What is needed is a way of conducting statistics that more intimately links theory to data, either via inference by intervals or via Bayes factors. Using canned statistical solutions to force theorizing is backward; statistics are to be the handmaiden of science, not science the butler boy of statistics.

Bayes has its own coherent logic that makes it radically different from significance testing. To be clear, a two-step decision process is not being advocated in this paper whereby Bayes is only performed after a non-significant result. Bayes can – and should – be performed any time a theory is being tested. At this stage of statistical practice, however, orthodox statistics typically need to be performed for papers to be accepted in journals. In cases where orthodoxy fails to provide an answer, Bayes can be very useful. The most common case where orthodoxy fails is where non-significant results are obtained. But there are other cases. For example, when a reviewer asks for more subjects to be run, and the original results were already tested at the 5% level, Bayesian statistics are needed (as in the Tan et al. (2014) example above; orthodoxy is ruled out by its own logic in this case). Running a Bayes factor when non-significant results are obtained is simply a way that we as a community can come to know Bayes, and to obtain answers where we need answers, and none are forthcoming from orthodoxy. Once there is a communal competence in interpreting Bayes, frequentist orthodox statistics may cease to be conducted at all (or maybe just in cases where no substantial theory exists, and no prior constraints exists – that is, in impoverished pre-scientific environments).

One complaint about the approach presented here could be the following: “I am used to producing statistics that are independent of my theory. Traditionally, we could agree on the statistics, whatever our theories. Now the statistical result depends on specifying what my theory predicts. That means I might have to think about mechanisms by which the theory works, relate those mechanisms to previous data, argue with peers about how theory relates to different experiments, connect theory to predictions in ways that peers may argue about. All of this may produce considerable discussion before we can determine which if any theory has been supported!” The solution to this problem is the problem itself.

Bayes can be criticized because it relies on “priors,” and it may be difficult to specify what those priors are. Priors figure in Bayesian reasoning in two ways. The basic Bayesian schema can be represented as: Posterior odds in two theories = Bayes factor × prior odds in two theories. The prior odds in two theories are a sort of prior. The approach illustrated in this paper has lifted the Bayes factor out of that context and treated it alone as a measure of strength of evidence (cf. Royall, 1997; Rouder et al., 2009). So there is no need to specify that sort of prior. But the Bayes factor itself requires specifying what the theories predict, and this is also called a prior. Hopefully it is obvious that if one wants to know how much evidence supports a theory, one has to know what the theory predicts.

The approach in this paper, though Bayesian, involves approaching analysis in a different way in detail than one would if one followed, say, Kruschke (2010a) or Lee and Wagenmakers (2014), who also define themselves as teaching the Bayesian approach. There are many ways of being a Bayesian and they are not exclusive. The philosophy of the approach here, as also illustrated in my papers published to date using Bayes, is to make the minimal changes to current practice in the simplest way that would still bring many of the advantages of Bayesian inference. In many cases, orthodox and Bayesian analyses will actually agree (e.g., Simonsohn, unpublished, which is reassuring, even to Bayesians). A key place where they disagree is in the interpretation of non-significant results. In practice, orthodox statistics have not been used in a way that justifies any conclusion. So one strategy, at least initially, is to continue to use orthodox statistics, but also introduce Bayesian statistics simultaneously. Wherever non-significant results arise from which one wants to draw any conclusion, a Bayes factor can be employed to disambiguate, as illustrated in this paper (or a credibility or other interval, if minima can be simply established). In that way reviewers and readers get to see the analyses they know how to interpret, and the Bayes provides extra information where orthodoxy does not provide any answer. Thus, people can get used to Bayes and its proper use gradually debated and established. There need be no sudden wholesale replacement of conventional analyses. Thus, you do not need to wait for the revolution; your very next paper can use Bayes.

The approach in this paper also misses out on many of the advantages of Bayes. For example, Bayesian hierarchical modeling can be invaluable (Kruschke, 2010a; Lee and Wagenmakers, 2014). Indeed, it can be readily combined with the approach in this paper. That is, the use of priors in the sense not used here can aid data analysis in important ways. Further, the advantages of Bayes go well beyond the interpretation of non-significant results (e.g., Dienes, 2011). This paper is just a small part of a larger research program. But the problem it addresses has been an Achilles heel of conventional practice (Oakes, 1986; Wagenmakers, 2007). From now on, all editors, reviewers and authors can decide: whenever a non-significant result is used to draw any conclusion, it must be justified by either inference by intervals or measures of relative evidence. Or else you remain silent.

## Conflict of Interest Statement

The author declares that the research was conducted in the absence of any commercial or financial relationships that could be construed as a potential conflict of interest.

## Footnotes

1 Typical for psychology is no more than 50%. Cohen (1962), Sedlmeier and Gigerenzer (1989); see Button et al. (2013), for a considerably lower recent estimate in some disciplines.

2 Rule (i) is especially relevant for testing the assumptions of a statistical model. Take the assumption of normality in statistical tests. The crucial question is not whether the departure from normality is non-significant; but rather, whether whatever departure as exists is within such bounds that it does not threaten the integrity of the inferential test used. For example, is the skewness of the data within such bounds such that when the actual alpha rate (significance) is 5% the estimated rate is between 4.5% and 5.5%? Or is the skewness of the data within such bounds such that a Bayes factor calculated assuming normality lies between 2.9 and 3.1 if the Bayes factor calculated on the true model is 3? See Simonsohn et al. (unpublished) for an example of interval logic in model testing. Using intervals inferentially would be a simple yet considerable advance on current practice in checking assumptions and model fit.

3 If inferences are drawn using Bayesian intervals (e.g., Kruschke, 2010a), then it is rules i and ii that must be followed. The rule of rejecting the null when a point null lies outside the credibility interval has no basis in Bayesian inference (a point value always has 0 probability on a standard credibility interval); correspondingly, such a rule is sensitive to factors that are inferentially irrelevant such as the stopping rule and intentions of the experimenter (Mayo, 1996; Dienes, 2008, 2011). Rules (i) and (ii) are not sensitive to stopping rule (given interval width is not much more than that of the null region; cf Kruschke, 2013b). Indeed, they can be used by orthodox statisticians, who can thereby gain many of the benefits of Bayes (though see Lindley, 1972; and Kruschke, 2010b; for the advantages of going fully Bayesian when it comes to using intervals, and Dienes, 2008, for a discussion of the conceptual commitments made by using the different sorts of intervals).

4 **Figure 1** represents the decisions as black and white, in order to be consistent with orthodox statistics. For Bayesians, inferences can be graded. Kruschke (2013c) recommends specifying the degree to which the Bayesian credibility interval is contained within null regions of different widths so people with different null regions can make their own decisions. Nonetheless, pragmatically a conclusion needs to be reached and so a good reason still need to be specified for accepting any one of those regions.

5 Bayes factors were independently discovered at about the same time by Harold Jeffreys et al. (1939/1961) and Alan Turing, the latter in order to help decode German messages during World War II (McGrayne, 2012).

6 Jeffreys et al. (1939/1961) also recommended labeling Bayes factors greater than 10 or less than 1/10 as “strong” evidence. However, the terms “substantial” and “strong” have little to choose between them. Thus Lee and Wagenmakers (2014) recommend calling Bayes factors greater than 3 or less than a 1/3 as “moderate” and those greater than 10 or less than 1/10 as “strong.”

7 Note this is not the same as specifying a 1- tailed or 2-tailed test in orthodox statistics. In orthodox statistics, conducting a 1- or 2-tailed test is a matter of whether one would treat a result as significant if the difference had been extreme in just one direction (1-tailed test) or rather in either direction (2-tailed test). Such counterfactuals do not apply to Bayes, and the criticisms of 1-tailed tests in an orthodox sense (criticizing a researcher using a 1-tailed test because he would have rejected the null if the results had been extreme in the other direction, even though they were not) hence do not apply to Bayes factors (cf. Royall, 1997; Dienes, 2011). Further, as shown in **Figure 3**, a two-tailed normal distribution can be used to make a directional prediction. So 1- vs 2-tailed for the Bayes factor just refers to the shape of the distribution used to represent the predictions of the alternative.

8 To have them scale in a similar way as a *t*-value, one could take a log Bayes factor to base 3. A log3 *B* above 1 indicates substantial evidence for the alternative, below -1 substantial evidence for the null, and 0 indicates no evidence either way. The value of 1024 in **Table 1** would become 6.31, which maybe seems more “reasonable.” Using log to base square root 3 would mean 2 and –2 are the cut offs for substantial evidence, similar to a *t*-test. Compare Edwards (1992), who recommended natural logs for Bayes factors comparing simple hypotheses. Nonetheless, I do not use log *B* in this paper. Unlike *t*, *B* has an intuitive meaning: The data are *B* times more likely under H1 than under H0.

9 Indeed, the absence of sharp cut-offs applies to Bayesian intervals as much to Bayes factors: If a 90% Bayesian interval were within the null region, there would be grounds for asserting the null region hypothesis, albeit there would be stronger grounds if a 95% interval were in the null region.

10 Using SD = mean/2 is like using the sampling distribution of the mean when it is only just significantly different from 0.

11 When a null region can be specified, Bayesian intervals and Bayes factors can be related (cf Kruschke, 2013b Appendix D; Wetzels et al., 2009, for a somewhat different way of making the relationship). Let the alternative hypothesis be the complement of the null region. Define a prior distribution. The prior odds is the area representing the alternative divided by the area in the null region. Update the prior to obtain the posterior distribution (see e.g., Kruschke, 2013b, or tools on the website for Dienes, 2008: http://www.lifesci.sussex.ac.uk/home/Zoltan_Dienes/inference/bayes_normalposterior.swf). The strength of evidence that the data provide for the alternative hypothesis over the null is the degree to which the evidence changes the prior odds: The Bayes factor is the posterior odds divided by the prior odds. Example: A prior of *N*(0, 1) and data of *N*(0.5, 0.2), i.e., mean = 0.5, SE = 0.2, gives a posterior (using the software on the website of Dienes, 2008, to obtain posteriors: http://www.lifesci.sussex.ac.uk/home/Zoltan_Dienes/inference/bayes_normalposterior.swf) of *N*(0.48, 0.2). For a null region of [-0.1, 0.1], using areas under the normal curve, we get *B* = 36.17/11.55 = 3.13. (This is not using the Dienes, 2008, Bayes factor calculator, just areas under the normal, with e.g., http://davidmlane.com/hyperstat/z_table.html). Conversely, a prior of *N*(0, 1) and data of (0, 0.1) give a posterior of *N*(0, 0.1) and *B* = 0.19. (Check these numbers for homework.)
