# Supplementary material for: Using Bayes to get the most out of non-significant results
Source: Front Psychol. 2014 Jul 29;5:781. doi: 10.3389/fpsyg.2014.00781 (PMC4114196; doi:10.3389/fpsyg.2014.00781)
Supplement: Supplementary file 1 [file Presentation_2.PDF]

Appendix 1 Further examples

1. *Trend analysis and other contrasts*. Parris and Dienes (2013) investigated whether the strength of a perceptual response (measured in milliseconds) to an imaginative suggestion in a non-hypnotic context varied with hypnotisability (measured on a 0 to 12 scale). Hypnosis researchers often call people scoring 0-3 as lows, 4-7 as mediums, and 8-12 as highs. Parris and Dienes recruited an equal number of lows, mediums and highs. A key question is the shape of the relation between hypnotisability and performance on the non-hypnotic task. On the one hand, it could be simply linear. On the other hand, it may be that highs are an unusual group, with mediums performing the same as lows; or that lows are an unusual group with mediums performing the same as highs (a question posed by Kirsch, 2011). The question can be answered by whether, in addition to a linear effect of hypnotisability on the task, there is a quadratic effect. Parris and Dienes regressed performance (in milliseconds) on hypnotisability (as a 0-12 scale) and obtained a raw slope of b = 6 milliseconds/unit hypnotisability, $t(33) = 2.261$, $p = .030$. To obtain the quadratic effect, the hypnotisability score was squared and centred on zero. Performance was regressed on both hypnotisability and hypnotisability squared. The slope for the quadratic effect was $b = .530$ milliseconds/unit hypnotisability^2, $t(33) = .589$, $p = 0.56$. Does the non-significant quadratic effect indicate that performance varies only linearly with hypnotisability?

The maximum plausible score of mediums is that of highs; thus, the minimum plausible quadratic effect would occur when mediums scored the same as highs, with lows scoring differently as a special group. Conversely, the maximum plausible quadratic effect would occur when mediums scored the same as lows, with highs scoring differently as a special group. To determine values for these extremes, first the mean performance for highs was found, and this mean set as the same performance for all mediums and highs; similarly, the mean for lows was set as the performance for all lows. This defined an idealized set of population values that were extreme in degree of quadratic effect. With this set of values treated as real data, the mean quadratic regression slope was - 0.673. Similarly, another hypothetical set of population vales was obtained by setting the mean of the lows as the same score for all lows and mediums; the highs all had the mean of the highs. With these values treated as data, the mean quadratic regression slope was 0.908. Thus, a Bayes factor can be determined assuming a uniform from -0.673 to +0.908. The mean for the data (i.e. in this case, the quadratic slope) is .53. It has a standard error of .53/.589 = 0.9. This gives a Bayes factor of 0.97. That is, the data were insensitive and provide no evidence either for or against the quadratic hypothesis. It would be wrong to use the non-significant quadratic effect to infer that mediums lay mid-way between highs and lows.

The principle for constructing limits for a uniform can be generalized. For a contrast, use values of the dependent variable in each condition that would represent extremes which are plausible given the scientific context. Coefficients found using the hypothetical scores as real data determine the plausible extremes of the contrast and can be used in a uniform to represent the alternative.

2. *Equating one variable to test the effect of another.* Song, Maniscalco, Koizumi, and Lau (2013) wished to investigate the function of confidence when performance was controlled. Thus, they found a way to create stimuli for which performance (d') was calibrated to be similar and yet confidence differed. We will call this performance single task d' (for reasons that will become clear) . (In what follows the numbers have been made up, so as not to jeopardise the publication of the authors' paper.) On a 1-4 confidence scale, the confidence for the two conditions was 2.21 and 2.41, $t(35) = 4.04$, $p = .0001$. The

corresponding single task d’s for the two conditions were 1.83 and 1.93, $t(35) = 0.96$, $p = 0.34$. The question is, can equivalence be claimed for single task d’ across the two conditions?

To allow comparability between confidence and single task d’, standardized effect sizes might seem useful. Then, it could be argued, the sort of effect size we might expect to see in single task d’, if there were an effect, would be in the same ball park as the standardized effect size found for confidence. We will use Pearson’s r as a standardized effect size, where $r^2 = t^2/(t^2 + \text{df})$. Thus, for confidence $r = \sqrt{(4.04^2/(35 + 4.04^2))} = .56$; and for d’, $r = \sqrt{(0.96^2/(35 + 0.96^2))} = .16$. Pearson’s r can be transformed to a normal variable with Fishers $z = 0.5*\log_e((1 + r)/(1 - r))$, which has standard error= $1/\sqrt{(\text{df} -1)}$. Thus, Fisher’s z for confidence = 0.64, and for d’ Fisher’s $z = 0.16$. The standard error for the Fisher’s z for d’ is 0.17. Thus, a Bayes factor can be determined for a mean of 0.16, SE = 0.17, and with the alternative represented as a half-normal with standard deviation equal to 0.64. In this case, $B = 0.64$. That is, the data appear insensitive for establishing equivalence as B is between 3 and 1/3.

However, the use of standardized effect sizes in this context doesn’t address the question of interest. A standardized effect size reflects the consistency with which an effect is shown, and thus how noisily it is measured (Baguley, 2009). One could make the measurement of performance noisy simply by using few trials. (Theoretically irrelevant differences in noise between measures occur for reasons other than just number of trials. Some measures are simply noisier than others: For example, confidence has a restricted range, d’ an unrestricted range.) By using a sufficiently small number of trials for performance, the population r for the difference between conditions in single task d’ could be made very small. If confidence was measured on a different large number of trials, its population r could be made large. That is, a researcher making the measure of single task d’ as insensitive as possible would help establish equivalence in single task d’ between conditions. This is also true for testing equivalence merely by showing a non-significant result. That is, both significance testing and Bayes using standardized effect sizes, do not address the real issue, in this case, because they are sensitive to factors that are theoretically irrelevant (and could therefore be cynically manipulated by the experimenter). To address the actual theoretical concerns of the researchers, raw effect sizes should be used.

But, how can one use a difference in confidence units to set a difference in d’ units? The researchers were interested in the extent to which confidence predicted a third outcome variable, specifically, discrimination on a task that involved integrating over many stimuli. Call this multi-task d’. That is, the most important issue was given that the difference in confidence was associated with a change in multi-task d’, could the difference in single task d’s be large enough to explain the change in multi-task d’? Given this theoretical agenda, we can calibrate confidence and single task d’ in the following way. The difference in confidence was associated with a significant difference in multi-task d’ in their experiment (in fact, this was the main result of the study). Let us say multi-task d’ differed by 0.6 units. So, let us say the researchers run another study in which single task d’ was allowed to vary over a range from say 1.5 to 2.5, and multi-task d’ was measured. Regress single task d’ on multi-task d’. Let us say the raw regression slope is 1.5 single d’ units/multi d’ units. Thus, the change in single task d’ that corresponds to 0.6 units change in multi-task d’ is 1.5*0.6 = 0.9. Now, we can use that estimated relevant change in single task d’ as the SD of a half-normal to represent a meaningful alternative to contrast with the null hypothesis of no difference in single task d’. For the Bayes factor calculator, the “mean” is 1.93-1.83 = 0.10 single task d’ units, the standard error is 0.10/0.96 = 0.10 single task d’ units, and we can use

a half-normal with an SD of 0.90 single task d’ units. In this case, $B = 0.31$, indicating we can sensitively accept the claim of equivalence.

This method finds the raw difference in d’ in a non-arbitrary way relevant to the scientific context. Further, there is no clear incentive to measure single task d’ insensitively. Making the measurement of single task d’ noisier (in an unbiased way) does not affect the expected raw regression slope of single task d’ against multi task d’. It just makes it harder to make sensitive claims when the Bayes factor on single task d’ is performed. (And making the measurement of multi-task d’ insensitive will reduce the slope, making equivalence claims harder.) A correct analysis and inference procedure will never be harmed by making data more sensitive.

Now let’s consider another possible method . Without knowledge of the slope of single task d’ against multi task d’, one could calibrate raw effect sizes by regressing single task d’ onto confidence and finding the raw slope. The difference in confidence was 2.41 - 2.21 = 0.2 confidence units. The raw slope was 0.45 d’ units/confidence unit, so the equivalent of 0.2 confidence units is 0.2*0.45 = 0.09 single task d’ units. Now, unlike with standardised effect sizes, making the measurement of d’ insensitive also does not help researchers trying to establish equivalence. As for the second method above, making the measurement of single task d’ more noisy (in an unbiased way) does not affect the raw regression slope. It just makes it harder to make sensitive claims. For the Bayes factor calculator, the “mean” is 1.93-1.83 = 0.10 single task d’ units, the standard error is 0.10/0.96 = 0.10, and we can use a half-normal with an SD of 0.09. In this case, $B = 1.39$, indicating insensitivity. We come to a different conclusion. So which method should be preferred, the second or the third? In fact, the second is most relevant in this scientific context. The third method assumes that the researchers were interested in differences in d’ only to the extent they were predicted by confidence - but the real point is the function of confidence itself in predicting multi-task d’. Note something peculiar about the third method: The smaller the relation between single task d’ and confidence, the smaller the estimate of single task d’ we are trying to pick up, and the harder it is to make claims of equivalence. But the extent of equivalence in single task d’ should be the same however confidence and single task d’ were correlated. Thus, the second method gets to the heart of the matter, not the third. The right statistical analysis depends on understanding the scientific problem. Canned statistical solutions are not the solution for statistics.

Up to now, we have talked about whether the claim of equivalence in single task d’ can be asserted. Whether or not the data are sensitive enough to support that claim, the claim that confidence predicts multi task d’ without being fully mediated by single task d’, can also be independently tested by mediation analyses. And in general the question of whether mediation is complete or whether there is no meditation at all are both claims that involve potentially accepting null hypotheses and thus (without specifying minimally interesting values) can only be answered by use of Bayes factors (see Semmens-Wheeler et al., 2013, for how to use the Dienes, 2008, Bayes calculator to give a quick and dirty answer (i.e. one that tests the product of regression slopes as if it satisfied the assumption of normality); Dienes, in preparation, for code that tests the null hypotheses in mediation analyses assuming only that estimates of individual raw regression slopes are normally distributed; and Nuijten, Wetzels, Matzke, Dolan, & Wagenmakers, submitted, for a different solution for obtaining Bayes factors for mediation analyses using standardized slopes.) By contrast, significance testing, including significance testing using Bayesian machinery to get the p-values (e.g. Yuan & MacKinnon, 2009), can obtain evidence for partial mediation but cannot in itself (without specification of minimally interesting values) allow assertion of either complete or no

mediation. Only a truly Bayesian mediation analysis can achieve the latter (presented in Dienes, in preparation, and Nuitjen et al, submitted).

3. Meta-analysis. Bayes factors can be used in meta-analysis to gain all the advantages of their use in single studies. Their use is most straightforward when the same paradigm has been repeatedly used. In this case, find the overall meta-analytic raw mean and overall meta-analytic standard error[1], and use these in the Bayes factor calculator. If standardized effect sizes are used to compare across studies with different paradigms and dependent variables, Pearson's r can be used as the standardized effect size, as r can be normalized with Fisher's *z*, with known standard error, and thus the Dienes (2008) calculator used. Bayes factors can in principle be multiplied together to accumulate evidence. However, in practice Bayes factors from single studies should not in general be multiplied together to get an overall Bayes factor, because this procedure does not take into account that initial studies should be used to update the alternative hypotheses of subsequent studies as they are added to the calculation in order to respect the axioms of probability (Jaynes, 2003). Thus, it is easier to first summarize the data and then determine the single Bayes factor on the summary, unless special precautions are taken (cf Rouder & Morey, 2011; Storm, Tressoldi, & Utts, 2013). (For point alternative hypotheses as used in the likelihood school of inference, e.g. Royall, 1997, the problem does not arise because if the alternative says only one point value is possible (e.g. the hypothesis that the population difference in means is exactly 10 seconds), the distribution representing the alternative – which is just a spike at the point value postulated - remains the same whatever the previous studies. Thus, in that case, Bayes factors can be un-problematically multiplied across single studies.)

4. Correlations and regression slopes. While correlations themselves are not normal, they can be made normal by a transformation, Fishers $z = 0.5*\log_e((1 + r)/(1 - r))$, which has standard error= $1/\sqrt{}$ (df -1). Thus, designs using correlations can make use of the Dienes (2008) Bayes calculator (see also Wetzels & Wagenmakers, 2012, for a default Bayesian analyses of correlations). However, in many cases, it is the raw regression slopes that really address theoretical concerns. Correlations may become reduced by a restriction of range or increased error in measurement of the dependent variable, but these factors do not affect the raw regression slope (which is nonetheless affected by error in measurement of the independent variable, as is a correlation). Raw linear regression slopes can be used with the Dienes (2008) calculator, just bearing in mind they have a standard error given by: raw slope/t.

5. Contingency tables. The following example illustrates the points:

1. One can interpret complex contingency tables by reducing to 1-df contrasts;

2. Researchers should be weaned off chi square tests of independence, which serve a purpose only for generating p-values and hence are essentially uninformative for non-significant results. Instead, one wants a measure of strength of association with known distribution and standard error;

---

[1] This can be achieved using software on the website for Dienes (2008) for finding a posterior from a prior and a likelihood. Entering the first study summary as the prior, the second study summary as the likelihood, and obtain the posterior mean and standard error; use the posterior as the prior for the next study, which is entered as the likelihood. Keep feeding in the posterior as the prior for the next study until all studies have been entered.

3. Ln OR (the natural log of the odds ratio) expresses strength of association and is normally distributed with known standard error;

4. Thus, having obtained Ln OR, one can play. One can compare strengths of association in different studies or conditions, put confidence intervals on the strength of association, and calculate Bayes factors to illuminate non-significant results.

McLatchie (2013) primed people in ways meant to cultivate guilty thoughts or guilty feelings. People subsequently given a sum of money then chose to either use it immediately as is, or to use it after a delay, when it would be increased; further, the use could be either to keep the money oneself, or else donate it to a good cause. One hypothesis was that guilty thoughts should increase pro-social behaviour (donating rather than keeping); another was that guilty feelings would increase impulsivity (using now rather than later). In Study 1 the following data shown in Table 4(a) were obtained.

Table 4

Data from Study 1 on Guilt Priming from McLatchie (2013)

(a) Full Data Set

	Keep Now	Keep Delay	Donate Now	Donate Delay
Guilty Feelings	6	2	7	5
Guilty Thoughts	3	2	1	14
Control	3	12	1	4

(b) A One-degree of Freedom Contrast

	Keep	Donate
Guilty Thoughts	5	15
Control	15	5

The data can be collapsed in a number of ways to answer specific questions. For example, to test if guilty thoughts increase pro-social action, a collapsed version of the data is shown in Table 4 (b). This yields a 2×2 Chi square test of independence of $\chi^2(1) = 7.69$, $p = .0056$. χ^2 does not directly express a magnitude of effect. One way of expressing the effect size is as an odds ratio: OR = (15*15)/(5*5) = 9.0. The OR is the product of cell counts along one diagonal, divided by the product along the other. If there is no association population OR = 1. We will put the diagonal on top which should be larger according to the theory, if the theory makes a directional prediction (as it does here). The natural log of the odds ratio, Ln (OR), is normally distributed with a squared standard error given by 1/A + 1/B + 1/C + 1/D, where A, B, C, D are each of the cell entries. So in this case, standard error = √

(1/5 + 1/15 + 1/15 + 1/5) = 0.73. Ln OR = Ln 9.0 = 2.20 in this case. We can test association with a *z*-test = 2.20/0.73 = 3.01, p = .0026[2].

In Study 2 using the same design but different methods of priming, Mclatchie obtained the data in Table 5(a), with the question of current interest shown in Table 5(b).

Table 5

Data from Study 2 on Guilt Priming from McLatchie (2013)

(a) Full Data Set

	Keep Now	Keep Delay	Donate Now	Donate Delay
Guilty Feelings	6	4	6	4
Guilty Thoughts	1	7	3	9
Control	2	14	7	17

(b) A One-degree of Freedom Contrast

	Keep	Donate
Guilty Thoughts	8	12
Control	16	24

Now the data look rather different. $\chi^2 = 0$ p = 1.00. More importantly, Ln OR = 0 with a standard error of 0.56. (So $z = 0$, $p = 1.00$.) Is the pattern significantly different in the second study? The Ln ORs differ by 2.20 – 0 = 2.20. The difference has a standard error given by the variance sum rule, namely squared standard error of difference is the sum of the squared standard errors. Thus standard error of difference = $\sqrt{(0.73^2 + 0.56^2)}$ = 0.92. Hence, to test the difference, z = 2.20/0.92 = 2.39, p = .017. Thus, the second study is a failure to replicate, the association is significantly less in the second rather than first study. Has the association been reduced to zero in the second study? The sort of effect size one might expect in the second study is provided by that in the first. Thus we can represent the alternative as a half-normal with SD = 2.20. The "mean" is 0, and the standard error is 0.56. This yields a B of 0.24, indicating substantial evidence for the null hypothesis of no association in the second study.

6. Comparing different theories. A Bayes factor compares theory 1 with theory 2. Call a Bayes factor comparing theory 1 with the null hypothesis $B_{1/0}$. Call a Bayes factor comparing theory 2 with the null hypothesis $B_{2/0}$. Then, the Bayes factor comparing theory 1 with theory 2, $B_{1/2} = B_{1/0} / B_{2/0}$ (Dienes, 2008). Thus, the Dienes (2008) Bayes factor calculator can be used to compare two substantial theories, or to compare a substantial theory to a null region hypothesis rather than a point null. For example, a uniform or a normal

[2] To obtain two-tail p-values for z, apart from using R, one can use online calculators, such as http://davidmlane.com/hyperstat/z_table.html: click on "outside" and enter – z and +z in the boxes.

defining a null region could be used (cf Morey & Rouder, 2011, who provide interesting discussion of the properties of such Bayes factors).

It is sometimes argued that a point null could never be really true (Cohen, 1994). But, it can be true to an astonishing degree of accuracy, given the scale of resolution of the data (Rouder et al, 2009). In this context, Baguley (2012, p. 369) cites a study on ESP with over 27,000 participants; the confidence interval for the proportion correct was [.496, .502], where 0.5 is the chance baseline. Thus, with well controlled and counter-balanced experiments using a point null hypothesis is not absurd, especially if a null region hypothesis is hard to specify. Nonetheless, null region hypotheses can also be entirely valid, for example where null regions are already decided in a literature (e.g. regarding changes of less than 3 units on the Hamilton depression scale as clinically uninteresting).

A Bayes factor could be used to compare the hypothesis that the change increased versus decreased by e.g. having a half normal with SD = expected effect size going in either direction. For example, for a mean difference of 5 seconds and an expected effect size, should it exist of 10 seconds in either direction, first run the calculator with a half normal with SD = 10, and data mean = 5 (and obtain $B_{1/0}$) ; then, run again by setting the data mean as -5 (and obtain $B_{2/0}$) . Dividing the two Bayes factors gives a Bayes factors comparing the two theories of change in each direction ($B_{1/2}$). Bear in mind that if the null hypothesis of zero change is true, $B_{1/2}$ is not driven in any particular direction as data is collected but performs a random walk. Thus sooner or later it will provide strong evidence for one of the two directional theories.

Appendix 2 Alternatives approaches for indicating relative evidence.

Likelihood inference: An alternative method to specifying a range of values predicted by the theory is to specify a single value. The likelihood school of inference recommends Bayes factors contrasting hypotheses of point values (Dienes, 2008; Royall, 1997). For example, one could compare the null hypothesis that the difference is 0 to an alternative that the difference is 10. In this way, the need to specify a distribution over population values is obviated. The Bayes factor will, in the limit as increasing observations are collected, come to favour the hypothesis that is closer to the truth. Thus, if the true population value is less than 5, eventually the null hypothesis will be supported over the alternative. If the population value is more than 5, the alternative will come to be supported. But, this means that 5 comes to function as a minimally interesting value. Thus, if one were to use point hypotheses in one's Bayes factor, one should decide on the minimally interesting value, m, and use an alternative point value of 2m.

In likelihood inference, there is no need to restrict oneself to a single Bayes factor. One could consider the relative strength of evidence for the full range of possible population values and thus construct a likelihood interval (Royall, 1997; see the Dienes, 2008, associated website for likelihood interval calculators). In this case, the rules of inference by interval apply, as shown in Figure 2. Thus, in general, likelihood inference contrasts with full Bayesian inference in requiring a specification of the minimally interesting effect size in order to connect data to theory.

BIC,AIC, DIC, etc. A range of model comparison techniques have been developed, often based on rather different principles, but all comparing the evidence for two models in a way that can be decomposed in terms of the model's degree of fit to the data, corrected by the number of free parameters in the model if this differs between models (see Baguley, 2012, chapter 11; Burnham & Anderson, 2002; Glover & Dixon, 2004; Wagenmakers, 2007). The main contrast with the approach illustrated in this paper is that such methods, when used for null hypothesis testing (which is not their only use), amount to assuming a vague default alternative hypothesis to compare against the null. This interpretation is most clear for *BIC*. (*AIC* is a measure of relative strength of evidence with in effect a default alternative for null hypothesis testing, one somewhat less vague than for *BIC*; Smith & Spiegelhalter, 1980.)

Wagenmakers (2007) showed that *BIC* approximates a Bayes factor that uses a "unit information prior" for the alternative (see Rouder et al., 2009, for further development of this specification to define vague defaults for Bayes factors). That is, the alternative is specified as a normal with the same mean as the mean of the data and a standard deviation equal to the standard deviation of the data. This can be justified on the grounds that if one had any accurate prior information at all, it would tend to specify the same mean as the data do, even if vaguely. Further, assuming the prior information was worth only one observation of data (hence "unit information"), the standard error of that information would be the standard deviation of the population data divided by square root of N, the number of observations – and N is just one. (Technically, one cannot use that very aspect of the data one is trying to predict – say its mean – to specify the alternative predicting the same data, as this involves "double counting", Jaynes 2003; however, the vagueness of the unit information prior renders the technical point harmless. One can also just set the mean of the alternative to zero to strictly deal with this point.) The *BIC* – or, correspondingly, the unit information prior used to specify the alternative with the Dienes (2008) Bayes calculator – may be worth considering if one finds it difficult to specify the alternative, or wishes to test a vast number of alternatives in a data mining operation. But, the responsibility still rests with the researcher

to consider seriously whether the unit information prior matches theoretical expectations. A vague prior makes finding evidence for the null easy (Kruschke, 2013b).

Consider trying to find evidence for unconscious processing by showing null performance on a test of awareness. It would seem prudent not to use a vague default, if only on the grounds that one wants to make the demonstration of unconscious knowledge convincing, and hence not especially easy to obtain. In fact, more importantly, Dienes (in press) shows in this situation that there are informative specifications defining the extent to which one expects conscious knowledge, in raw units, rendering defaults irrelevant in that scientific context.

Every case has to be considered on its own merits as to whether the specification of the alternative is relevant (Kruschke, 2013b; Vanpaemel, 2010). In that sense, true defaults (i.e. those that could be used at any time to avoid consideration of scientific context) do not actually exist in real scientific contexts. A Bayes factor is a means for comparing two theories. In the absence of theory, placing credibility or likelihood intervals around parameter estimates (without accepting or rejecting any null hypothesis) lets the data speak for themselves (Kruschke, 2013b).

Appendix 3 Robustness checking

The Bayes factor is sensitive to how vague H1 is specified as being; the vaguer H1, the more the data will support H0. The vagueness of H1 depends mainly on the rough maximum value allowed; however, in detail *B* can depend on the exact shape of the specified distribution as well. This raises a question: There may be a number of ways of specifying essentially the same scientific judgments. For example, the scientific judgment for H1 might simply be that the maximum population difference cannot be more than about 8. The exact shape for the distribution is not further specified; assigning a specific shape then adds more precision than the science in the situation actually provides. If *B* were sensitive to the shape beyond the specification of the maximum, the extent to which that *B* was relevant to the scientific theory could be questioned. On the other hand, if the decision based on *B* were robust to major shape changes in simple distributions given the same maximum, then *B* shows its relevance to evaluating the theory.

Here we consider several Bayes factors, subscripted to indicate the distributions used to represent H1 (thanks to Wolf Vanpaemal for this notational suggestion). $B_{N(m,sd)}$ means a normal was used with mean m and standard deviation sd; $B_{H(0, sd)}$ indicates a half-normal was used with standard deviation sd; $B_{U[min,max]}$ indicates a uniform was used between a minimum of min and a maximum of max. Letting a rough maximum for a normal correspond to two standard deviations out, B_N, B_H and B_U can then be set to respecting the same scientific intuition concerning the maximum population difference.

The Rouder Bayes factors are also shown below. $B_{JZS(r)}$ is the JZS *B* with scaling factor r, and $B_{UI(r)}$ uses the unit information prior with scaling factor r. B_{JZS} and B_{UI} can be taken to represent theories about Cohen's *d* rather than about raw mean differences so may produce somewhat different answers than B_N, B_H or B_U.

Consider a study where N = 30, the mean is 5.5 units above a null value of 0, SD = 13.7, so SE = 2.5. A predicted effect of about 5 units can be argued for; or a maximum of 10 units. The Rouder *B*'s were scaled with a predicted Cohen's *d* of 5/13.7 = 0.36. The first two rows of Table 6 illustrate a significant effect, and the B_N and B_U just tip over 3, as expected. Notice the distributions for the predictions of H1 are centred on 0, so the scientific theory in question predicts effects in either direction. In these cases it doesn't matter if the distribution is peaked in the middle (a normal) or flat (a uniform); the same conclusions follow. The Rouder *B*'s, scaled according to the Cohen's *d* of the expected effect size, produce somewhat less evidence for H1 than the corresponding B_N and B_U: B_{JZS} and B_{UI} represent theories about Cohen's *d* rather than about raw mean differences. B_H is rather higher than the others in the first row; this is because it represents a different scientific theory, namely a theory that predicts effects in only one direction. It is desirable that *B* may be different when different scientific theories are represented. The second row illustrates how a directional theory is penalized when the mean goes in the wrong direction.

Table 6

Different Specifications of H1

Mean	SE	$B_{H(0, 5)}$	$B_{N(0, 5)}$	$B_{U[-10,10]}$	$B_{JZS(0.36)}$	$B_{UI(0.36)}$
5.5	2.5	6.05	3.10	3.40	1.99	2.77
-5.5	2.5	0.15	3.10	3.40	1.99	2.77
0	2.5	0.45	0.45	0.31	0.34	0.45

Table 7

B's for a Directional Theory

Mean	SE	$B_{H(0, 5)}$	$B_{N(5, 2.5)}$	$B_{U[0,10]}$
5	2.5	4.27	5.22	4.42
-5	2.5	0.16	0.10	0.11
6	2.5	8.81	12.10	10.46
-6	2.5	0.14	0.10	0.09
0	2.5	0.45	0.26	0.31

Table 7 presents results for three different ways of representing a directional theory. As can be seen, the different distributions produce similar *B*'s, no matter whether the distribution is flat, peaked in the middle or pushed to one end. Thus, conclusions based on these *B*s will often be robust. In any give case, one needs to check the robustness of the conclusions. When evidence is near a threshold, different representations of H1 might tip either side of it (though bear in mind that the threshold is itself not meaningful for Bayes, it is just a convention).

Verhagen and Wagenmakers (in press) provide a different Bayes factor calculator that takes as its theory that the current study is an exact replication of a previous one, so H1 can be set as predicting the standardized effect size previously obtained, with an uncertainty defined by the previous standard error in the estimate. Their *B* closes matches the results obtained with the defaults recommended in this paper. For example, Verhagen and Wagenmaker's example 1 is based on Eliot et al (2010): The latter's experiment 2 was designed as replication of their experiment 1. In experiment 1 the raw effect size was 6.79 - 5.67 = 1.12 units for females. To interpret experiment 2, the 1.12 can be used as the SD of a half-normal. In experiment 2 of Eliot et al, a difference of about 1 raw unit (from the graph in the original paper) can be estimated for females; with a *t* of 3, this gives a standard error of 0.33. $B_{H(0, 1.12)}$ = 38.57, very close to the 39.73 obtained by Verhagen and Wagenmakers. For

males an SE of 0.33 can be estimated from the graph; thus, mean difference = SE*t = - .02. $B_{H(0, 1.12)}$ = 0.27, reasonably close to the 0.13 obtained by Verhagen and Wagenmakers. (Similar *B*s for the Dienes calculator and Verhagen and Wagenmakers calculator hold for their other examples.) In sum, the different *B*s, based on somewhat different distributions, but the same scientific intuitions, produced reassuring close answers, indicating the robustness of the conclusions.

In sum, the often close results obtained by different Bs when the same scientific theories are represented validates *B*s as a method of evaluating such theories. The arbitrariness of the distributions chosen to represent H1 can be shown to be inconsequential when different simple distributions representing the same theories produce qualitatively the same answers. When different answers are obtained for representations of H1 that are equally plausible as representations of the same scientific judgments, more data need to be collected until the conclusion is robust.
